# Seasonal Fluctuations in Iron Cycling in Thawing Permafrost Peatlands

**DOI:** 10.1021/acs.est.1c06937

**Published:** 2022-03-15

**Authors:** Monique S. Patzner, Nora Kainz, Erik Lundin, Maximilian Barczok, Chelsea Smith, Elizabeth Herndon, Lauren Kinsman-Costello, Stefan Fischer, Daniel Straub, Sara Kleindienst, Andreas Kappler, Casey Bryce

**Affiliations:** † Geomicrobiology, Center for Applied Geosciences, University of Tuebingen, Schnarrenbergstrasse 94-96, 72076 Tuebingen, Germany; ‡ Abisko Scientific Research Station, Swedish Polar Research Secretariat, Vetenskapens väg 38, SE-891 07 Abisko, Sweden; § Department of Geology, Kent State University, Kent, Ohio 44242, United States; ∥ Department of Biological Sciences, Kent State University, Kent, Ohio 44242, United States; ⊥ Environmental Sciences Division, Oak Ridge National Laboratory, 1 Bethel Valley Road, Oak Ridge, Tennessee 37830, United States; # Tuebingen Structural Microscopy Core Facility, Center for Applied Geosciences, University Tuebingen, Schnarrenbergstrasse 94-96, 72076 Tuebingen, Germany; ∇ Microbial Ecology, Center for Applied Geosciences, University Tuebingen, Schnarrenbergstrasse 94-96, 72076 Tuebingen, Germany; ○ Quantitative Biology Center (QBiC), University Tuebingen, Auf der Morgenstelle 10, 72076 Tuebingen, Germany; ◆ Cluster of Excellence: EXC 2124: Controlling Microbes to Fight Infection, 72074 Tübingen, Germany; ¶ School of Earth Sciences, University of Bristol, Bristol BS8 1RJ, U.K.

**Keywords:** soil organic carbon, iron, bioavailability, permafrost collapse, seasonal fluctuations, microbial Fe(III) reduction and Fe(II) oxidation, Abisko, Arctic

## Abstract

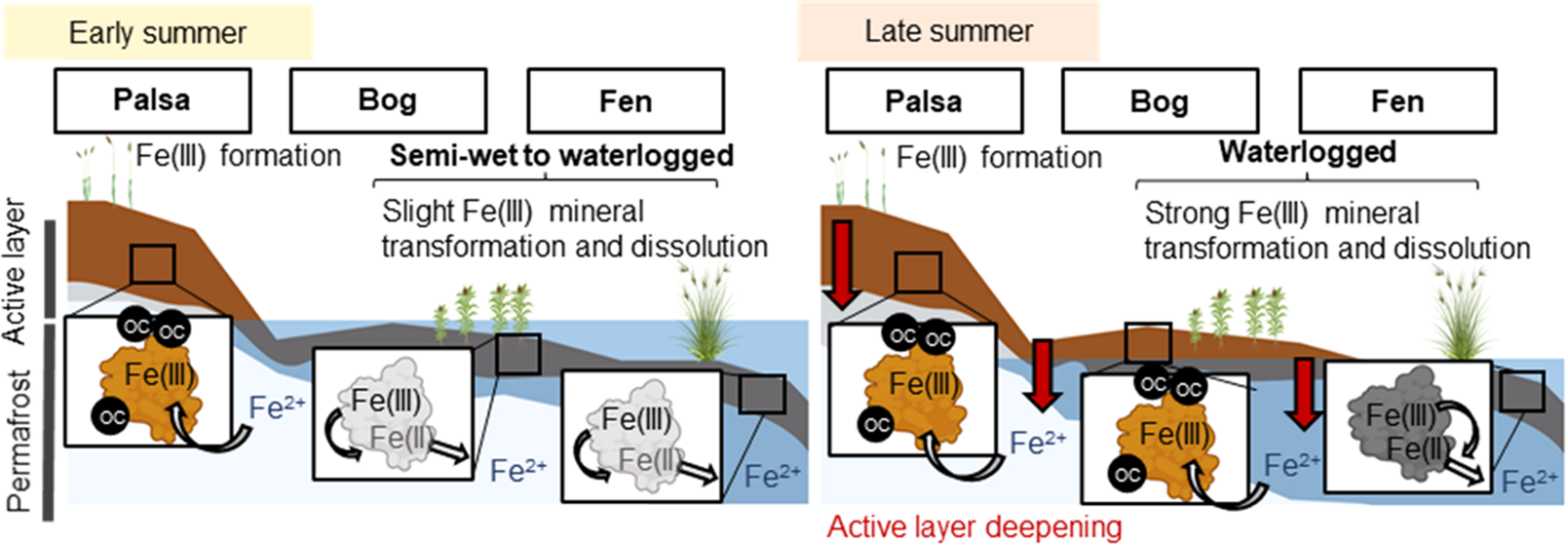

In permafrost peatlands, up to 20% of total organic carbon (OC) is bound to reactive iron (Fe) minerals in the active layer overlying intact permafrost, potentially protecting OC from microbial degradation and transformation into greenhouse gases (GHG) such as CO_2_ and CH_4_. During the summer, shifts in runoff and soil moisture influence redox conditions and therefore the balance of Fe oxidation and reduction. Whether reactive iron minerals could act as a stable sink for carbon or whether they are continuously dissolved and reprecipitated during redox shifts remains unknown. We deployed bags of synthetic ferrihydrite (FH)-coated sand in the active layer along a permafrost thaw gradient in Stordalen mire (Abisko, Sweden) over the summer (June to September) to capture changes in redox conditions and quantify the formation and dissolution of reactive Fe(III) (oxyhydr)oxides. We found that the bags accumulated Fe(III) under constant oxic conditions in areas overlying intact permafrost over the full summer season. In contrast, in fully thawed areas, conditions were continuously anoxic, and by late summer, 50.4 ± 12.8% of the original Fe(III) (oxyhydr)oxides were lost via dissolution. Periodic redox shifts (from 0 to +300 mV) were observed over the summer season in the partially thawed areas. This resulted in the dissolution and loss of 47.2 ± 20.3% of initial Fe(III) (oxyhydr)oxides when conditions are wetter and more reduced, and new formation of Fe(III) minerals (33.7 ± 8.6% gain in comparison to initial Fe) in the late summer under more dry and oxic conditions, which also led to the sequestration of Fe-bound organic carbon. Our data suggest that there is seasonal turnover of iron minerals in partially thawed permafrost peatlands, but that a fraction of the Fe pool remains stable even under continuously anoxic conditions.

## Abstract 2

Reactive iron minerals trap or release significant amounts of organic carbon depending on seasonal redox shifts in partially thawed permafrost peatlands.

## Introduction

Permafrost peatlands hold enormous amounts of organic carbon (OC), equivalent to over one-third of the carbon currently in the atmosphere (∼800 Pg).^1,2^ By the end of this century, permafrost peatlands are predicted to warm from an annual average air temperature below 2 °C to between +5.6 and +12.4 °C,^3^ almost twice the rate of the global average.^4^ The resultant permafrost thaw leads to soil active layer deepening,^5,6^ changes in surface vegetation composition,^7,8^ altered carbon accumulation,^2,9^ and shifts in microbial communities that degrade or transform OC.^10−12^ Ultimately, permafrost peatlands are shifting from a carbon sink to a source of greenhouse gases (GHG).^1,13,14^ What controls how fast and to what extent this will occur is currently intensively studied.

One parameter relevant for controlling GHG emissions in permafrost environments could be the protection of OC by Fe minerals. Fe–OC associations regulate long-term global preservation of natural organic matter in soils and sediments, and potentially also in the active layer underlain by intact permafrost and in partially thawed soils.^15,16^ Patzner et al.^15^ demonstrated that substantial quantities of carbon are trapped by iron minerals in regions with intact permafrost. With permafrost thaw, this mineral-bound OC is mobilized by reductive dissolution of Fe(III) minerals promoted by Fe(III)-reducing bacteria under water-logged and oxygen-limited conditions.^15,17^ The resulting dissolved OC (DOC) can then be further metabolized and can lead to GHG emissions during permafrost thaw.^18^

However, permafrost peatlands not only experience long-term change (i.e., over yearly or decadal timescales) but also seasonal changes in freeze–thaw cycles,^19^ air temperature,^20^ sunlight,^21^ and precipitation.^20^ With seasonal shifts in runoff and soil moisture,^20^ soils could drain and (re-)flood.^22^ Redox conditions, which are controlled by the presence of electron acceptors such as O_2_, fluctuate between oxic (oxygen-rich, drained) and anoxic (oxygen-depleted, flooded) conditions, where alternative electron acceptors (e.g., Fe(III), NO_3_^–^, SO_4_^2–^, CO_2_) are converted to reduced species (e.g., Fe(II), NO_2_^–^/N_2_, H_2_S, CH_4_). Hence, these seasonal redox changes either promote or suppress Fe(III) reduction and Fe(II) oxidation, in turn influencing carbon mineralization and ultimately, GHG emissions.^23−25^ Fe-associated OC in these environments are dominated by short-range-ordered co-precipitates^15^ and are thus highly vulnerable to dissolution under anoxic conditions. However, the regularity with which they are recycled by Fe(III) reduction and Fe(II) oxidation during seasonal redox fluctuations, or whether there is some potential for longer-term stability, is unknown.

To capture the spatial and temporal dynamics of iron cycling over the thawed summer season from July to September, we coated sand with synthetic Fe(III) oxyhydroxide minerals (ferrihydrite; FH; simplified formula of Fe(OH)_3_), filled it into porous Teflon bags, and placed them in the active layer at three regions of a Swedish permafrost peatland (Stordalen mire, Abisko) that differ in thaw severity. All bags were deployed at the same time and collected either in early summer (after 2 week incubation; July) or in late summer (after 2 months; September collection). The discrete thaw stages in which the bags of FH-coated sand were deployed were (1) desiccating palsa underlain by intact permafrost, (2) partially thawed bog that shows evidence of permafrost collapse, and (3) fully thawed fen with no remaining permafrost.^11,18,26,27^ The objectives of this study were (i) to quantify Fe(III) (oxyhydr)oxide formation, dissolution, and transformation and (ii) to quantify Fe-associated OC trapped by the bags along the thaw gradient as redox conditions shift in the thawed summer season.

## Materials and Methods

### Field Site

Stordalen mire in the Abisko region of northern Sweden is a degrading permafrost peatland.^28,29^ Increasing mean annual air temperatures, exceeding the 0 °C threshold, led to rapid warming of the Abisko region since the 20th century^30^ causing active layer deepening and an increase in surface wetness through thawing of permafrost.^5^ As previously described,^11,15,18^ the mire consists of three distinct forms of degrading permafrost peatland: (1) palsas which overlie intact permafrost and are generally oxic and well draining, (2) partially thawed ombrotrophic bogs that are water-logged, acidic, and dominated by *Sphagnum* spp., sedges and shrubs, and (3) minerotrophic, permafrost-free fen that is heavily water-logged, influenced by circumneutral pH groundwater and dominated by sedges, mainly *Eriophorum* spp.^26^ (Figure S1).

### Experimental Design

To capture the spatial and temporal dynamics of iron cycling over the thawed summer season from July to September, we incubated bags containing sand coated with synthetic Fe(III) oxyhydroxides (here FH) in the Stordalen mire peatland. Bags were deployed in the active layer of the palsa, bog, and fen along the permafrost thaw gradient described above^15,18^ (for FH-coated sand synthesis and bag preparation, see the Supporting Information (SI), as well as Figures S1 and S2). To compare seasonality in the response of the iron cycle, we performed both short (2 weeks) and long (2 months) incubation times. All bags were deployed at the same time in June 2019 and then incubated for either 2 weeks (collected in early July 2019) or for 2 months (collected in September 2019). For the short-term incubation, three bags each were placed at three sites in each of the three thaw stages (nine bags per thaw stage in total; for exact positions, see Figure S1). For the longer incubation, three bags each were deployed at each of the three thaw stages (three bags per thaw stage, nine in total; Figure S1). We refer to the short-term treatment as the “early summer” bags, and the longer-term treatment as the “late summer” bags. To install the bags, the first 10 cm of the soil layer were removed with a coring sleeve and the bags placed into the hole, which was then sealed again with the upper 10 cm soil. Upon collection, the FH bags were carefully taken out of the soil, immediately frozen in liquid nitrogen, and stored at −80 °C until further analysis. Before soil extractions, further geochemical and microbial community analysis, the FH-coated sand that was incubated in different bags at the same thaw stage site (palsa, bog, and fen) and collected at the same time point was homogenized to have enough sample material to optimize the methods and to avoid limited sample volume for replicate analysis. The reported values represent the average and standard deviation of triplicate analysis of nine homogenized bags per thaw stage for the short-term incubation and three homogenized bags per thaw stage for the long-term incubation (Figure S1). A portion of the dried FH-coated sand was set aside to be used as a reference material. This was stored alongside the experimental bags until their deployment (including during transport to Sweden), and stored in the dark at room temperature until the field experiment was complete. When the experimental bags were collected, the reference bags were then frozen alongside them until analysis.

### Sequential Fe Extractions

Sequential Fe extractions were used to follow changes in solid-phase Fe transformation along the thaw gradient over the season. Anoxic Na-acetate solution (1 M, pH 5), followed by extractions with 0.5 and 6 M HCl were used to successively dissolve Fe phases with increasing crystallinity.^31^ Adsorbed Fe(II)^32,33^ and Fe in amorphous Fe sulfides^34^ were extracted by the Na-acetate (referred to adsorbed/amorphous Fe). 0.5 M HCl was chosen to extract poorly crystalline Fe(III) (oxyhydr)oxides and remaining reduced Fe(II) species such as FeCO_3_^35^ or FeS (referred to as poorly crystalline Fe) and 6 M HCl to extract more crystalline, remaining Fe fractions, such as more crystalline Fe(III) (oxyhydr)oxides, poorly reactive sheet silicate Fe or FeS species^15,31^ (referred to as more crystalline Fe) from the Fe mineral coated sand (for the exact extraction procedure, see the SI). Total Fe is calculated as the sum of Fe extracted by 1 M Na-acetate and 0.5 and 6 M HCl. Values obtained from the experimental bags were compared to those obtained from the reference sand to determine how incubation in the soil had altered the abundance and crystallinity of the Fe phases in the bags.

### Total Organic Carbon (TOC) Analysis

To quantify the TOC content of the sands after incubation, triplicate samples of the homogenized samples (all bags per thaw stages and incubation times) were dried at 60 °C until no further weight loss was observed. The samples were ground to fine powders and analyzed by a SoilTOC instrument (Elementar Analysensysteme GmbH, Germany).

### Determination of OC Bound to the Fe Mineral Surface

A sodium pyrophosphate extraction (pH 10) was used to remove loosely bound OC. This includes labile and microbial OC as well as OC in Fe–OC complexes and Fe–OC colloids.^15,36−38^ Sodium pyrophosphate solubilizes organic matter (of labile OC and microbial origin), dissolves Fe from organic complexes, and promotes peptization and dispersion of Fe oxide colloids which makes it difficult to specify the source of extracted Fe and OC.^39,40^ The same amounts of homogenized sand and extract were used as for the sequential Fe extraction, only the incubation time was extended to 16 h, as previously suggested.^15,36^ The Fe extracted by the sodium pyrophosphate extraction, representing colloidal/OM-chelated Fe, was negligible ((0.00 ± 0.00)−(0.18 ± 0.07) mg sodium pyrophosphate extractable Fe per g sand) in comparison to the total Fe ((1.02 ± 0.09)–(5.59 ± 0.46) mg Fe per g sand, 0.04–18.06% of the total Fe). The OC extracted by the sodium pyrophosphate extraction is implicated in complex association structures, such as colloids or aggregates, but not mineral-bound OC.^36,41^ Given (i) that the bags consisted only of FH and quartz, and (ii) that crystalline quartz such as that present in the bags has low potential for complexation with organic compounds,^42^ we assume that all of the carbon quantified via TOC of the FH coated sands after subtracting loosely bound OC (sodium pyrophosphate extractable OC) is strongly associated with the present Fe minerals (eq 1)
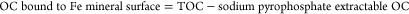
1

### Geochemical Analyses

Extracts (supernatants) were analyzed in analytical triplicates for Fe (extracted by sodium acetate, 0.5 or 6 M HCl or sodium pyrophosphate) and OC (extracted by sodium pyrophosphate).^15^ Extracts for Fe analysis were immediately stabilized under anoxic conditions in 1 M HCl dilutions. Fe(II) and Fe(tot) were determined by the spectrophotometric Ferrozine assay^43^ within 24 h. Fe(III) was calculated by subtraction of Fe(II) from Fe(tot). DOC was quantified in triplicate with a TOC analyzer (High TOC II, Elementar, Elementar Analysensysteme GmbH, Germany). Inorganic carbon was removed by the addition of 50 μL of 2 M HCl to 1.5 mL of samples prior to analysis.

### Scanning Electron Microscopy (SEM) and Energy-Dispersive X-ray Analysis (EDS)

These analyses were conducted on the reference sand and a portion of the homogenized sand that had been incubated in the palsa, bog, or fen for 2 months as described in the SI.

### Microbial Community Analysis

Sand from three replicate bags representing each thaw stage and exposure time was homogenized and total DNA extracted as described previously.^44^ Briefly, the PowerSoil RNA and DNA isolation kit was used to extract DNA in triplicates with the following modifications: 2 g of sand was used from each bag; beat-beating was conducted for 10 min and centrifugation was at maximum speed (7,000*g*) at 4 °C. During extractions, the incubation time was extended to 1.5 h at −20 °C (for details, see the SI). Library preparation steps and sequencing were performed by Microsynth AG (Switzerland) as detailed in the SI. Quality control, reconstruction of 16S rRNA gene sequences, and taxonomic annotation were performed with nf-core/ampliseq v1.1.2,^45,46^ as outlined in the SI.

Isolation of Fe(III)-reducing bacteria was performed with anoxic synthetic fresh water media (as previously described^15^) using a dilution to extinction approach (for further information, see the SI).

### Seasonal Geochemical Monitoring

To capture seasonal fluctuations in weather and soil geochemical conditions, context data such as precipitation, air temperature, soil moisture, and soil temperature were analyzed. Precipitation and air temperature data were provided by the Abisko Observatory. Soil moisture and soil temperature data were provided by Integrated Carbon Observation System (ICOS) Sweden Abisko—Stordalen.^47^ Redox potentials in the palsa, bog, and fen were continuously monitored with five redox potential probes (PaleoTerra). Two probes were positioned in both the bog and fen and one was positioned in the palsa. Each redox probe had platinum sensors positioned at 6, 8, and 10 cm depth below the ground surface (for details, see the SI).

## Results and Discussion

### Seasonal Fluctuations Drive Redox Shifts in Thawing Permafrost Peatlands

**Figure 1:**
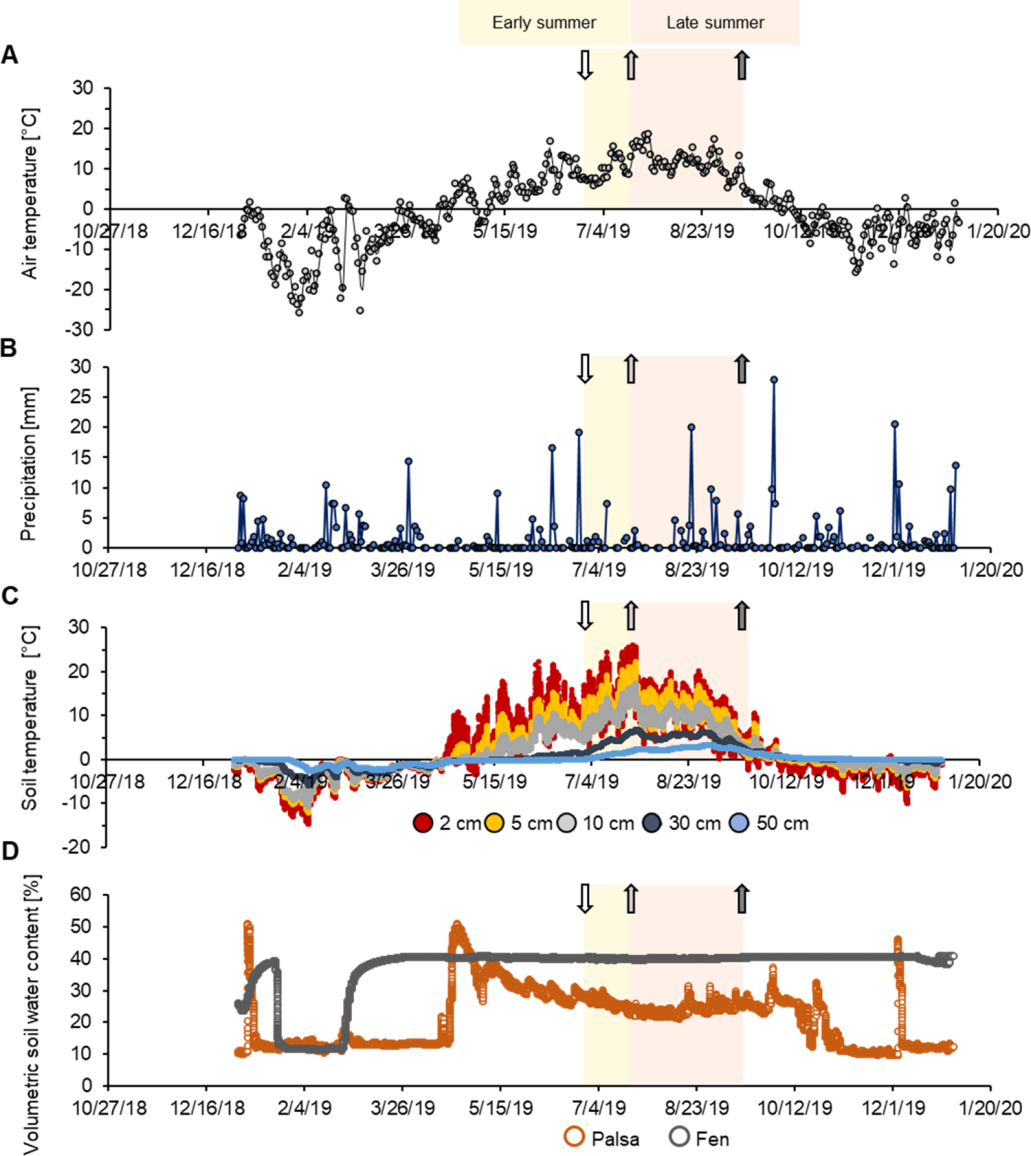
Seasonal fluctuations in weather and soil conditions for Abisko and Stordalen mire in the year 2019. (A) Air temperature [°C] and (B) precipitation [mm] were monitored by the Abisko Observatory. (C) Average soil temperature at Stordalen mire (average of the three thaw stages palsa, bog, and fen) at 2, 5, 10, 30, and 50 cm depth and (D) volumetric soil water content [%] in the upper 6 cm from the soil surface in palsa and fen were monitored by Integrated Carbon Observation System (ICOS) Sweden Abisko—Stordalen.^47^ For thaw stage-specific soil temperatures, see Figure S6. Early summer (yellow background) marks the time period when the short-term ferrihydrite (FH) bags were deployed for 2 weeks. “Late summer” bags (red background) were deployed at the same time as the early summer bags but remained in the soil for 2 months. The white arrow marks start of short- and long-term incubations. The light gray arrow marks the end of short-term deployment (only capturing early summer), and the dark gray arrow marks the end of the long-term deployment (deployed from early to late summer).

Snow melt began in the second half of April in 2019 (air temperatures above 0 °C, Figure 1) and lasted around 1 month. As previously shown, the meltwater resulted in the highest annual runoff (up to 75% of the total annual runoff).^20^ This was reflected in our own data set by the volumetric soil water content (VSWC) which peaked at 51% in the intact palsa between the end of March and the beginning of April (Figure 1), after which it declined and remained low. The influx of meltwater presumably results in increasing runoff into the partially thawed bog^48^ which would decrease as the palsa dried. This is reflected in the redox data from the bog. In early summer, semiwet bog soils (pH ∼ 4) were weakly (+100 to +300 mV^47^) to moderately (−100 to +100 mV^49^) reduced from 6 to 10 cm depth (Figures 1 and S1). From the beginning of the thawed season (soil temperatures above 0 °C in May/June), the air temperature increased to a maximum of 18.7 °C, accompanied by a soil temperature increase to a maximum of 25.0 °C in 2 cm soil depth at the end of July (Figure 1). Increasing evapotranspiration, together with decreasing runoff from intact palsa and increasing active layer depth (30–70 cm),^20,22,26^ likely contributed to soil drainage in the partially thawed bog (Figures 1 and S4). Ultimately, a shift from weakly/moderately reduced to oxic conditions (redox potential above +300 mV) in late summer was observed (Figures 1 and S5).

Through the whole thaw season, the palsa remained relatively dry and oxic (Figure S5), whereas the fen stayed water-logged (VSWC of 40%, Figure 1) and weakly to moderately reduced (Figure S5), confirming previous studies.^18,50^ The annual average air temperature of +0.2 °C slightly exceeded the 0 °C threshold (above 0 °C ice melts and permafrost thaws) supporting the overall warming trend since the early 20th century.^30^ The summer of 2019 was dry: only 60 mm rain fell in June and July (Figure 1) compared to the long-term average of 81 mm (1913–2009).^20^

### Fe(III) Mineral Formation and Dissolution under Changing Redox Conditions

**Figure 2:**
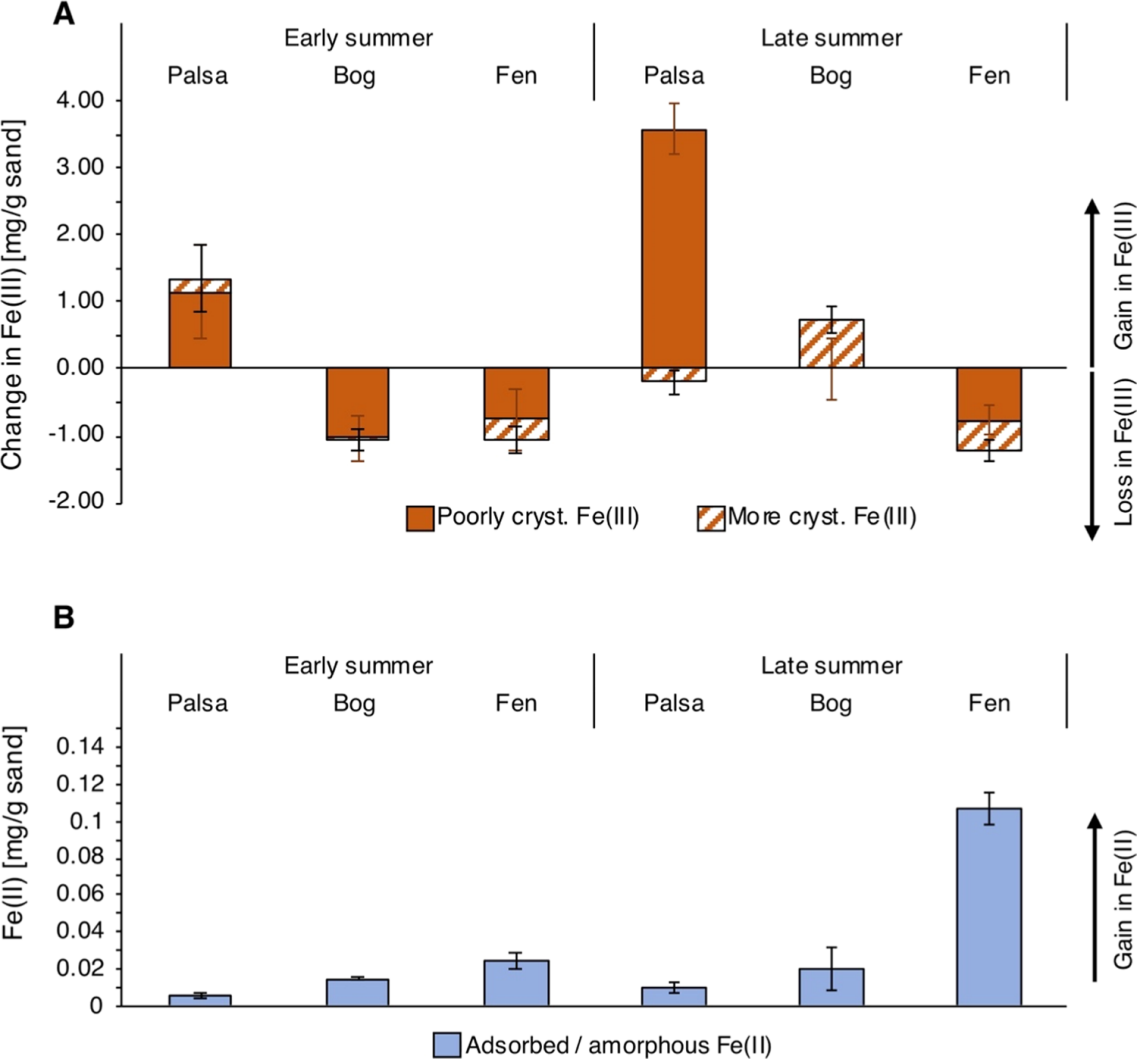
Gain and loss of solid-phase iron (Fe) along a thaw gradient in early (2 week incubation) and until late summer (2 month incubation). (A) Gain and loss in poorly crystalline Fe(III) (0.5 M HCl extractable) and more crystalline Fe(III) (6 M HCl extractable). Values are normalized to the reference material (unexposed ferrihydrite (FH)-coated sand with 2.19 ± 0.26 mg total Fe per g sand), which was transported to the field but then stored at room temperature until the end of the experiment. The reference material included a more crystalline Fe phase (1.01 ± 0.14 mg only 6 M HCl extractable per g sand), probably due to aging over time. Positive values indicate a net gain in Fe, and negative values indicate a net loss in Fe in comparison to the reference material. (B) Adsorbed/amorphous Fe(II) (1 M Na-acetate extractable). No Fe(III) was detected in the 1 M Na-acetate extracts. Reported values are the average of triplicate analysis, normalized to the reference material, of sand homogenized from all bags deployed at each thaw stage (palsa, bog, and fen). Error bars are the combined standard deviation of the triplicate analysis. Nine bags per thaw stage were combined from the early summer collection, and three bags per thaw stage were combined for the late summer collection.

In the active layer of the palsa underlain by intact permafrost, continuous oxic conditions promoted Fe(II) oxidation to Fe(III) phases through early to late summer, presumably from the influx and oxidation of dissolved Fe(II) from the surrounding soil which has a porewater Fe(II) concentration of up to approximately 2 mM.^77^ A (62.1 ± 40.2)–(155.3 ± 27.3)% gain in solid Fe(III) (0.5 and 6 M HCl extractable) was observed in bags deployed in early to late summer ((3.55 ± 0.87)–(5.58 ± 0.44) mg Fe(III) per g sand in comparison to 2.19 ± 0.26 mg per g sand in the reference material). In the active layer of the partially thawed bog, weakly to moderately reduced redox conditions in early summer favored Fe(III) (oxyhydr)oxide reduction which is indicated by Fe(III) mineral dissolution leading to a 47.2 ± 20.3% loss of Fe (i.e., loss of 1.03 ± 0.34 mg 0.5 M HCl extractable Fe(III) per g sand, Figure 2). However, a shift to predominantly oxic conditions in the bog in the late summer, caused by seasonal redox fluctuations, promoted net Fe(II) oxidation, indicated by a 33.7 ± 8.6% gain in Fe(III) in the bags that remained in the soil until late summer relative to the reference material. The newly formed Fe phases were more crystalline, as indicated by a gain in 0.73 ± 0.21 mg of 6 M HCl extractable Fe per g sand in comparison to the reference material (that contained 1.01 ± 0.14 mg of 6 M HCl extractable Fe per g sand) probably due to aging over time (Figure 2). Constant dissolution and reprecipitation have been shown to promote the formation of more crystalline oxides in previous work.^24,58,59^

In the active layer of the fully thawed fen, continuous weakly to moderately reducing conditions led to substantial Fe loss and slight Fe(II) accumulation through early to late summer. 50.4 ± 12.8% Fe(III) was lost from the solid phase (0.98 ± 0.27 mg Fe(III) remaining) and 0.11 ± 0.01 mg Fe(II) per g sand (1 M Na-acetate extractable) were formed during exposure.

The gain of poorly crystalline Fe in bags deployed in the active layer of intact palsa, attributed to Fe(II) oxidation under oxic conditions, supports previous observations showing highest amounts of Fe(III) (oxyhydr)oxides at the redox interface between shallow organic and deeper mineral horizons within the seasonally thawed active layer overlying intact permafrost.^15,51^ Fe(III) formation during late summer, as observed in bags deployed in the active layer of the partially thawed bog, could explain the presence of reactive Fe(III) phases (i.e., sodium dithionite citrate extractable iron^52^) in bog soils: Patzner et al.^15^ found that 7.5% of the 6 M HCl extractable iron in partially thawed bog soils was reactive Fe(III). The newly formed, more crystalline Fe(III) phase in bags deployed in the bog until late summer could be explained by the exposure of poorly crystalline Fe(III) oxyhydroxide minerals to microbially derived Fe(II) which can induce mineral recrystallization and transformation processes of Fe(III) oxyhydroxides towards thermodynamically more stable mineral phases.^53^ Along the thaw gradient, aqueous Fe(II) in the porewater increased from 0.02 ± 0.01 mM in the palsa to up to 1.6 ± 0.3 mM in the fen^15^ and the pH from 4.1 in the bog to 5.8 in the fen.^18^ Fe(II)-catalyzed transformation of FH can result in either goethite (α-FeOOH), lepidocrocite (γ-FeOOH), or magnetite (Fe_3_O_4_) formation with only minor differences in pH, background ligands, and Fe(II)/Fe(III) ratios impacting reaction kinetics and end-phase.^53−57^ The presence of DOC in the partially thawed bog^15,18^ could also cause mineral transformation to less-crystalline FH and lepidocrocite through precipitation of Fe-OC co-precipitates.^58^ FH on the sand grains could also trigger a so-called template effect similar to that observed by Chen and Thompson^59^ who saw that goethite formation in various forest soils was promoted by new minerals using pre-existing Fe(III) (oxyhydr)oxides as a template for crystallization. The nonextractable mineral phase (here: quartz) has only a minor effect on oxidation rates.^59^ Further studies are needed to identify the newly formed Fe phases by, e.g., Mössbauer spectroscopy or X-ray diffraction (XRD) analysis, which was not possible for this experimental setup as we could not effectively separate enough Fe from the sand grains for these analyses.

Our incubation experiments provide a mechanistic explanation for the porewater Fe(II) concentrations observed before in fen soil at Stordalen mire (Sweden)^15^ and in other Arctic peat soils in Barrow (Alaska).^60^ Lipson et al.^61^ estimated that net reduction of Fe(III) (oxyhydr)oxides coupled to oxidation and mineralization of OC contributes to 40–63% of ecosystem respiration depending on organic layer thickness and season. In the bags containing FH-coated sand, Fe(III) reduction was most likely driven by Fe(III)-reducing bacteria such as *Geobacter* spp., demonstrated by the fact we successfully isolated Fe(III)-reducing bacteria from the FH-coated sand in bags deployed in the fen with a 16S rRNA gene sequence that shares 98% identity to *Geobacter* spp. In addition, *Geobacter* spp. comprised 0.45 ± 0.01% relative abundance in 16S rRNA gene amplicon sequencing of the whole microbial community associated with sand deployed in the fully thawed fen until late summer (see Figures S7 and S8).

We observed incomplete Fe(III) reduction and dissolution in bags deployed in the bog and fen, resulting in 52.5 ± 20.3% loss of the initial Fe(III) in the bog in early summer and 50.4 ± 12.8% loss of the initial Fe(III) in the fen through the whole summer. The remaining Fe(III) phase in our experiments might explain the presence of small quantities of reactive Fe(III) phases (2.64 ± 0.03 mg Fe per g soil) at the redox boundary between organic and mineral horizon in the fully thawed fen, suggesting a minor but persistent Fe fraction remaining in soils even with complete permafrost thaw.^15^ This incomplete Fe(III) reduction and dissolution could have a number of explanations: (1) The Teflon bag itself may slow Fe(III) reduction rates probably due to slightly limited access for bacteria and hydrophobicity of the Teflon: in Teflon packed FH-coated sands, Fe(III) reduction rates were slightly lower than in unpacked FH-coated sand (Figure S9), (2) FH-coated sand could have a lower susceptibility to reductive dissolution compared to aluminum-silicate-FH co-precipitates,^62^ which are typically present in soils, (3) Remaining Fe(III) minerals are less accessible for microbial Fe(III) reduction due to the formation of Fe(II)-surface coatings, which lower the reducibility of Fe minerals.^41^ (4) The remaining Fe(III) phase could also be sustained by present Fe(II)-oxidizing bacteria such as *Gallionella* spp. and *Sideroxydans* spp. (Figures S7 and S8), although this seems to be unlikely due to very low dissolved O_2_ concentrations (0.15 ± 0.04 μM in partially thawed bog and 0.02 ± 0.01 μM in fully thawed fen in early summer, i.e., mid-July^50^).

The remaining Fe(III) phase could also be explained by net oxidation of Fe(II) even under reduced conditions. Previously, Lipson et al.^61^ observed net oxidation of Fe(II) in the active layer of reduced permafrost soils for which several hypotheses are suggested: (1) Fe(II) oxidation by O_2_ or by microaerophilic Fe(II)-oxidizers could be driven by transport of oxygen to deeper layers by plant roots,^63^ such as *Eriophorum* spp. at Stordalen mire, (2) high concentration of dissolved Fe in these soils (1.6 ± 0.3 mM aqueous Fe^2+ ^^15^) that might circulate throughout the soil profile, becoming oxidized to Fe(III) abiotically by O_2_ or by microaerophilic Fe(II)-oxidizers at the surface and diffusing to lower layers, (3) oxidation of Fe(II) under anoxic conditions e.g., by microbes coupled to nitrate reduction, abiotically by reactive N-species (e.g., nitrite), perchlorate reduction, by phototrophic Fe(II)-oxidizers or radicals formed by light;^64,65^ or (4) direct flow of e^–^ through conductive soil components such as metal (e.g., Fe) ions in the porewater and electric double layer of colloidal surfaces of organic matter and metal ions (highly abundant in peat soils^66^) that can couple anoxic processes at depth to oxic processes at the surface.^67^ One such conductivity component could even be caused by the presence of cable bacteria that link the oxidation of Fe(II) in anoxic layers to the reduction of O_2_ at the surface^44^ and/or *Geobacter* spp. (Figures S7 and S8) which produces conductive biofilms, pilin nanofilaments (known as microbial nanowires), and nanoparticulate Fe (oxyhydr)oxides^68,69^ that form conductive networks over centimeter scales with Fe(III)-reducing microbial cells^70^ and humic substances that can shuttle electrons to Fe(III) (oxyhydr)oxides.^71^

Previous work has shown that iron minerals effectively trap OC in intact permafrost soils, but that this is lost with complete permafrost thaw.^15^ Our new findings suggest a more dynamic microbial iron cycle in the intermediate, partially thawed bog, under seasonal fluctuations that can either promote or suppress Fe(II) oxidation and thus Fe(III) mineral formation.

### Carbon Accumulation by Fe Mineral Phases under Changing Redox Conditions in Thawing Permafrost Peatlands

**Figure 3:**
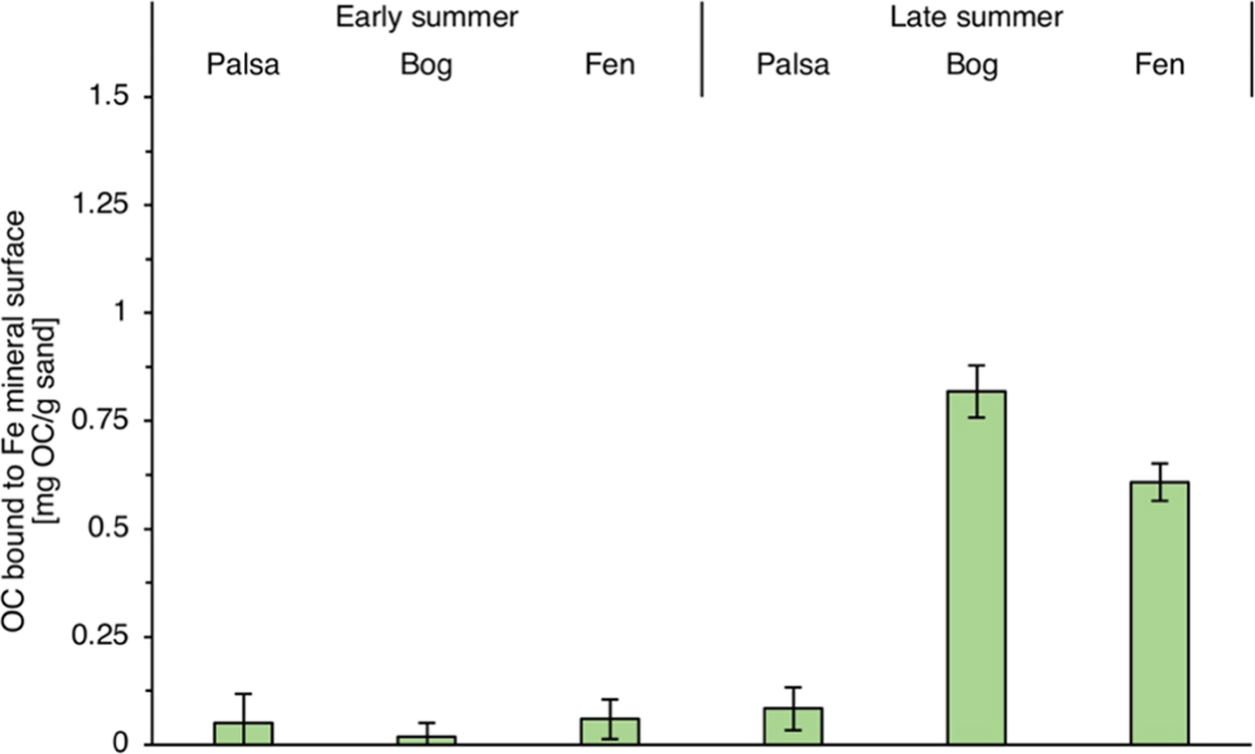
Organic carbon (OC) associated with iron (Fe) mineral phases along the thaw gradient following 2 week (early summer collection) and 2 month incubation in soil (late summer collection). Reported values represent the total OC control-corrected by subtracting loosely bound OC (sodium pyrophosphate extractable OC). Error bars represent the combined standard deviation of total OC and sodium pyrophosphate extractable OC.

**Figure 4:**
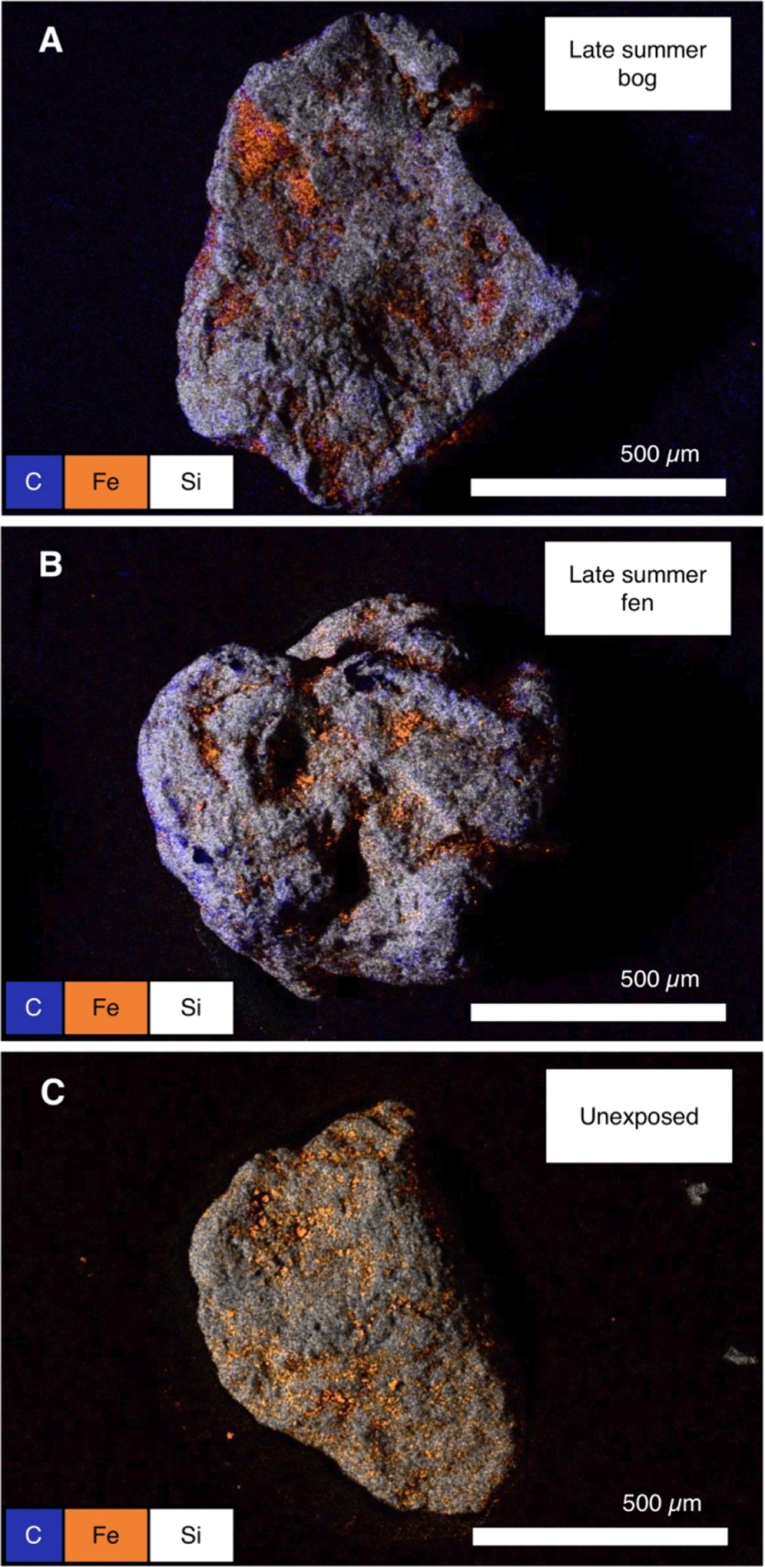
EDS-derived chemical distribution maps of iron (Fe)–organic carbon (OC) associations on ferrihydrite (FH)-coated sand grains incubated in the partially thawed bog (A) and in fully thawed fen (B) after 2 month incubation (late summer collection) in comparison to the reference material (unexposed FH-coated sand) (C). Results shown are representatives, and replicate analysis is reported in the Supporting Information (Figures S11 and S12). The orange color of the unexposed sand appears brighter than that of the samples retrieved from the bog and fen because of the co-occurrence with the purple color indicative of carbon.

In the active layer of palsa, almost no OC was associated with the Fe mineral phases after the period of early summer deployment (0.05 ± 0.07 mg OC per g sand) or after collection in the late summer (0.08 ± 0.04 mg OC per g sand) (Figure 3). In the bags that were only deployed for 2 weeks in early summer, almost no OC associated with the Fe mineral surface was observed in the bags from the partially thawed bog (0.02 ± 0.02 mg OC per g sand) and fully thawed fen areas (0.06 ± 0.03 mg OC per g sand) along the thaw gradient, probably due to the overall loss of Fe caused by mineral dissolution. However, in the bags collected after 2 months, carbon accumulation on the sand grains was observed in the active layer of the bog (0.82 ± 0.05 mg OC per g sand) and in the fen (0.61 ± 0.04 mg OC per g sand) at the end of the summer (Figure 3). OC/Fe (w/w) ratios were 0.28 in the bog and 0.73 in the fen at the end of the summer (see Table S1). These OC/Fe (w/w) ratios suggest co-precipitation with and/or chelation of metal (Fe) ions by organic compounds which can generate OC/Fe associates with C/Fe ratios (w/w) above 0.22.^16,72−74^ Microscopic images of Fe and C on the sand grain surface show evidence of co-occurring deposits of Fe and C on the surfaces of the sand (Figures 4 and S10–S12).

It should be noted that the FH-coated sands cannot only capture the mobile phase of OC (i.e., DOC) in the porewater but also particles smaller than 0.55 mm diameter, which is the size of the holes in the Teflon bags and thus would include particulate organic carbon and microbes. Along the thaw gradient, porewater DOC (i.e., aqueous C <0.22 μm) increases from 19.7 ± 0.8 mg/L in the palsa to 102.1 ± 14.1 mg/L in the fen areas^15^ and biomass increases with 2.6 times more microbial cells per gram of soil found in fully thawed fen relative to palsa and bog.^11^ The increasing microbial biomass along the thaw gradient may explain the observation of the highest sodium pyrophosphate extractable OC in bags that experienced long-term incubation in the bog (34.13% of TOC) and fen (38.12% of TOC) (Figure S3), and why our DNA extraction was only successful for bags deployed in fen until late summer (Figures S7 and S8).

Previously, it was shown that OC associated with reactive Fe minerals was higher in intact palsa soils than in other soils (bog and fen) along a permafrost thaw gradient,^15^ whereas here in our field incubation experiments, FH-coated sands placed in intact palsa soils did not sequester any OC onto the mineral surface. Dissolved Fe^2+^ in soils migrates upwards to the redox interface where it is oxidized to form Fe(III) (oxyhdyr)oxides or organic-bound Fe(III).^51^ This process leads to the observed increase of Fe in bags deployed in intact palsa soils, which we attribute primarily to Fe(III) (oxyhydr)oxide formation given the low abundance of pyrophosphate extractable Fe (Figure S3). DOC, however, is more dynamic than Fe as it might be metabolized and transformed to CO_2_ and CH_4_ prior to reaching the FH we experimentally incubated in the active layer underlain by intact permafrost. Chen et al.^24^ found dissolved organic matter (DOM) and soil organic matter (SOM) protection by Fe only under static oxic conditions when Fe^2+^ and DOC were both present. Whereas oxidation of Fe^2+^ without DOC promoted OC mineralization via fenton reactions.^24^ Abiotic oxidation of dissolved Fe^2+^ by O_2_ produces hydroxyl radicals that are known to degrade organic matter to low molecular weight organic molecules and CO_2_.^75,76^ Thus, the absence of porewater DOC, due to its potential high bioavailability,^77^ as well as the radical formation following abiotic oxidation of dissolved Fe^2+^ by O_2_ could explain the absence of OC associated with the FH-coated sands in the active layer of the palsa soils.

The highest carbon sequestration in these experiments occurred after Fe(II) oxidation and Fe(III) mineral formation in bags deployed in the active layer of the partially thawed bog until late summer and could explain previous observations that highest percentages of reactive Fe-bound OC were found in bog soils along the thaw gradient after palsa collapse (39.4% of TOC was reactive Fe-associated at the redox boundary between organic and mineral horizon).^15^ However, our data shows that the majority of the Fe-OC in the partially thawed bog is not stable over the course of the summer. These minerals go through periods dominated by Fe(III) reduction and others dominated by Fe(II) oxidation, with these iron redox transformations tightly linked to the ability of these minerals to accumulate Fe-OC

Accumulation of OC associated with the Fe mineral surface is also seen in the bags deployed in the fully thawed fen until late summer despite persistent anoxic conditions. The Fe(III) oxides remaining in these bags by late summer seem resistant to mineral reduction and dissolution and can capture OC from the surrounding porewater. The presence of remaining Fe(III) oxides in the FH bags after mineral reduction and dissolution could explain previous observations of small quantities of Fe(III)-OM (determined via extended X-ray absorption fine structure (EXAFS) and Fe(III)-citrate as reference probes) in fully thawed fen soils.^15^ However, it remains unclear, why, after mineral dissolution occurred, the Fe(III) mineral phases didn’t sequester small amounts of OC in the bags in the partially thawed bog and fully thawed fen after early summer incubation for 2 weeks. This could be explained by differences in the mineralogy of the remaining Fe(III) phase or Fe(II)-OC coatings on the sand grains surface in the fully thawed fen. The latter possibility is supported by the fact that the highest solid Fe(II) was observed in bags removed from the fully thawed fen in late summer and that the fen lacked any measurable reactive Fe-associated OC (i.e., associated with Fe(III)) in previous studies.^15^

Our data indicate that iron cycling in these soils likely promotes carbon dioxide release (by promoting Fe(III)-reduction coupled to OC mineralization) in early summer. However, Fe(II) oxidation in late summer in the partially thawed bog provides a potential mechanism for DOC immobilization (via formation of Fe (oxyhydr)oxide minerals and sequestration of associated OC). Thus, the influence of Fe on carbon cycling in these systems strongly depends on seasonal fluctuations in runoff, soil moisture, and ultimately, redox conditions.

### Environmental Implications

Permafrost environments experience drastic changes caused by climate change.^13^ Rising temperatures in the Arctic^78^ trigger increasing permafrost temperatures^79^ and ultimately an increase in the thickness and variability of the active layer.^80^ Multiple lines of evidence exist that the Arctic hydrological cycle is intensifying because of warming,^81^ leading to a rise in all fluxes including precipitation, runoff, and evapotranspiration.^82^ These changes ultimately drive rapid shifts in water levels and redox conditions from flooded and more reduced to drained and oxic permafrost-affected soils.^83^ The present study demonstrates that iron cycling in thawing permafrost peatlands correlates with redox conditions and that shifts in redox conditions resulted in either Fe(II) oxidation and Fe(III) mineral formation, sequestering OC, or leading to Fe(III) reduction, which would likely result in OC release. Iron cycling between Fe(II) and Fe(III) depending on shifts in redox conditions driven by seasonal fluctuations in runoff and soil moisture was also shown in a drained thaw lake basin on the Arctic coastal plain.^61^ This seasonality in the cycling of Fe and associated OC in permafrost environments has the potential to drive GHG emissions. On the one hand, Fe(III) reduction can lead to direct CO_2_ emissions since it is coupled to oxidation and mineralization of organic matter.^61,65^ Additionally, the released previously Fe-associated OC becomes more accessible to decomposers such as e.g., fermenters.^11^ Fe(III) reduction can also inhibit methanogenesis by being more thermodynamically favorable.^84^ McCalley et al.^10^ found seasonal variations in CH_4_ fluxes and their ^13^C content in partially thawed bog and fully thawed fen at Stordalen mire, which could be partly driven by the use of Fe(III) in microbial metabolisms. On the other hand, stable and newly formed Fe(III) minerals can sequester OC and protect it from microbial consumption,^85,86^ thus suppressing GHG emissions. The presence of minerals can play an important role in carbon release from permafrost soils. For example, Lee et al.^87^ observed nearly 20 times lower carbon release on a per gram soil basis via aerobic respiration in incubation experiments with permafrost-affected mineral soils in comparison to organic soils. This lower carbon release in mineral soils could be caused by mineral OC sequestration, although could also be influenced by the generally lower OC content of mineral soils. Additionally, Adhikari et al.^88^ demonstrated nearly 30% lower aerobic respiration of organic compounds sorbed to ferrihydrite directly demonstrating that the kinds of iron–carbon interactions we observe can directly influence greenhouse gas emissions.

Our data shows that reactive Fe minerals in the active layer of partially thawed bog soils in permafrost peatlands are largely unstable over the thawed summer and are continuously recycled by Fe(III) reduction and Fe(II) oxidation. Redox shifts likely result in iron minerals alternating between being an organic carbon source (i.e., in early summer when reducing conditions dominate) to a carbon sink (i.e., later in the summer when bags deployed in the bog are shown to accumulate carbon). Despite this rapid turnover, there also appears to be some fraction of iron minerals that remain resistant to dissolution during persistent anoxia (as demonstrated by bags deployed in the fen). This works highlights that future studies will be required to assess the extent of GHG emissions caused by the formation, transformation, and destruction of Fe(III) minerals under these fluctuating redox conditions in thawing permafrost environments.^89,90^

## Supporting Information Available

Sites for deployment of the bags with ferrihydrite-coated sand along the thaw gradient from palsa (intact) to bog (partially thawed) to fen (fully thawed) at Stordalen mire, Abisko (Sweden) (Figure S1); examples of ferrihydrite-coated sand in Teflon bags (Figure S2); sodium pyrophosphate extractable iron and organic carbon (Figure S3); seasonal changes at Stordalen mire in early summer and late summer (Figure S4); redox potential along the thaw gradient (Figure S5); soil temperatures at each thaw stage at different soil depths (Figure S6); microbial community of ferrihydrite-coated sand deployed for 2 months in the fully thawed fen until late summer (Figure S7); bacterial 16S rRNA gene copy numbers based on qPCR analysis (Figure S8); microbial Fe(III) reduction under lab conditions in loose ferrihydrite-coated sand versus ferrihydrite-coated sand in Teflon bags (Figure S9); SEM surface analysis of the ferrihydrite-coated sands before and after incubation (Figure S10); EDS-derived chemical distribution maps of the replicate analysis of iron and organic carbon (Figure S11); EDS-derived chemical distribution maps of iron coating on unexposed ferrihydrite-coated sand grains (Figure S12); and organic carbon (OC)-to-iron (Fe) ratios (Table S1) (PDF)

## Notes

Raw sequencing data were deposited at the Sequence Reads Archive (SRA, https://www.ncbi.nlm.nih.gov/bioproject/PRJNA769643). Other data are included in the Supporting Information.
