# Supplementary material for: Seasonal Fluctuations in Iron Cycling in Thawing Permafrost Peatlands
Source: Environ Sci Technol. 2022 Mar 15;56(7):4620–31. doi: 10.1021/acs.est.1c06937 (PMC9097474; doi:10.1021/acs.est.1c06937)
Supplement: Supplementary file 1 — es1c06937_si_001.pdf [file es1c06937_si_001.pdf]

Seasonal Fluctuations in Iron Cycling in Thawing Permafrost Peatlands

*Monique S. Patzner[1], Nora Kainz[1], Erik Lundin[2], Maximilian Barczok[3], Chelsea Smith[4], Elizabeth Herndon[5], Lauren Kinsman-Costello[4], Stefan Fischer[6], Daniel Straub[7,8], Sara Kleindienst[7], Andreas Kappler[1,9] & Casey Bryce[10]**

[1]Geomicrobiology, Center for Applied Geosciences, University of Tuebingen, Germany.

[2]Swedish Polar Research Secretariat, Abisko Scientific Research Station, Sweden.

[3]Department of Geology, Kent State University, US.

[4]Department of Biological Sciences, Kent State University, US.

[5]Environmental Sciences Division, Oak Ridge National Laboratory, US.

[6]Tuebingen Structural Microscopy Core Facility, Center for Applied Geosciences, University Tuebingen, Germany.

[7]Microbial Ecology, Center for Applied Geosciences, University Tuebingen, Germany.

[8]Quantitative Biology Center (QBiC), University Tuebingen, Germany.

[9]Cluster of Excellence: EXC 2124: Controlling Microbes to Fight Infection, Tübingen, Germany.

[10]School of Earth Sciences, University of Bristol, UK.

SUPPORTING INFORMATION

Number of figures in supporting information: 12

Number of tables in supporting information: 1

Total numbers of pages in supporting information: 23 (including cover page)

Synthesis of Ferrihydrite-coated Sand. Quartz sand with a grain size of 0.4-0.8 mm (Carl Roth GmbH + Co.KG, Germany) was used. The sand was pre-treated by first autoclaving the sand at 121°C for 20 minutes, followed by washing with 1 M HCl (24 h, completely covered) and finally with Aqua Regia for 5 min to further improve Fe coating efficiency by increasing the surface area, as has been done previously[1, 2]. Afterwards the sand was washed with MilliQ water and dried at 60°C.

The Fe(III) oxyhydroxide 2-line ferrihydrite (FH) was synthesized in the presence of the sand by precipitation from a $Fe(NO_3)_3*9H_2O$ solution by adding 1 M KOH[1]. The $Fe(NO_3)_3*9H_2O$ solution was added to 500 g of sand and stirred manually by hand as KOH was added dropwise until a pH of 7.3 was reached. The mixture was then left without stirring. After two hours, the pH was readjusted to 7.5 and the mixture left overnight on a rolling shaker (15 rpm), as has been done previously[1]. Finally, the mixture was washed with MilliQ water and dried at 40°C to avoid temperature-induced modifications of the precipitates[1].

In the end, the fresh synthesized ferrihydrite-coated sand yielded an iron content of 2.19±0.26 mg poorly crystalline Fe(III) per g sand, determined by 0.5 M HCl extraction, performed in triplicates, followed by Fe quantification in the extract by Ferrozine assay[3]. Treating the unexposed sand in the same manner as the sand incubated within the soils (i.e. transport to the field and back, and stored at room temperature), formed a more crystalline Fe(III) phase (1.01±0.14 mg Fe(III) per g sand), which was only extractable by 6 M HCl, not extractable with 1 M Na-acetate and 0.5 M HCl. As also previously stated[1], the FH coating increased the specific surface area (SSA) from 0.07 m^2 per g of the initial pure sand to 1.49 m^2 per g of the FH-coated sand (21 times higher than for its uncoated precursor).

Ferrihydrite Bags. The FH-coated sand was packed in Teflon bags (polytetrafluoroethylene (PTFE); or Teflon®) with 0.1 mm thickness and manually pierced with needles of 0.55 mm

diameter under sterile conditions in a Biological Safety Cabinet (BSC). The bag was closed with a cable tie at the top and with a long plastic line, which was later used to mark the position of the bag at the surface and pull it out after incubation. The Teflon along with additional equipment (FH coated sand, cable ties) was autoclaved (121°C, 1 bar pressure, 20 mins) prior to use, brought into the BSC cabinet and exposed to UV-light for 15 minutes. The Teflon was chosen based on the following suitable properties: heat (up to 250°C) and cold (until -196°C) resistant; unaffected by most chemicals, especially iron; no adhesive forces and weather.

The bags filled with FH-coated sand were stored at room temperature in sterile plastic bags for 3-4 weeks before being transported to the field under sterile conditions.

Sequential Fe Extraction. 0.5 g of homogenized sand from each thaw stage and collection periods was added under anoxic conditions (100% N_2, remaining O_2 <100 ppm) into Eppendorf tubes. As previously described[6], samples were centrifuged (5 min, 12,000 g) and the porewater (supernatant) was removed. 1 mL of anoxic 1 M Na-acetate solution was added to the pellet, mixed and incubated for 24 h in the dark. Then, the sample was centrifuged (5 min, 12,000 g) and the supernatant was collected and stored anoxically in the dark at 4°C until further analysis. To the pellet, 1 mL of anoxic 0.5 M HCl was added, mixed and incubated in the dark under anoxic conditions for 2 h. After centrifugation again, the supernatant was removed and stored anoxically at 4°C until further analysis. To the remaining pellet, 1 mL of anoxic 6 M HCl was added, mixed and incubated for 24 h in the dark as the last extraction step[6]. The supernatant was again stored under anoxic conditions in the dark at 4°C until further analysis.

Scanning Electron Microscopy (SEM) and Energy Dispersive X-Ray Analysis (EDS). SEM and EDS were acquired in order to visualize changes in Fe and C on the surface of the sand grains. The sand of the reference material (unexposed FH-coated sand) and the homogenized

sand from bags deployed for the longer 2 month incubation (palsa, bog and fen) were anoxically dried (24 h, 100% N_2 atmosphere). After reaching constant weight, the dried sand was pressed in an indium band (Plano GmbH, article number: E432), specifically chosen to avoid a carbon background signal in the EDS analysis. The indium band was then glued to the SEM sample stub with conductive silver paint (ACHESON Silver DAG 141; Plano GmbH, article number: G3692). After a short drying time (5 min), the samples were immediately coated with ~8 nm Pt-layer using a BAL-TEC SCD 500 sputter coater, operated in a working distance of 35 mm, for 90 seconds at 0.02 mbar. SEM and EDS analysis were performed at the Center for Applied Geosciences, University Tuebingen, using a Zeiss Crossbeam 550L Cryo-FIB-SEM, equipped with an Oxford EDS detector (UltimMax 100) and AZtecEnergy Advanced software. SEM images were acquired at a working distance of 5 mm by use of the Secondary Electrons Secondary Ions (SESI) detector using an acceleration voltage of 30 kV during the EDS analysis session and at 5 kV (for images about surface information) with a 39x, 250x and 650x magnification and a store resolution of 2048 pixel image width. Four replicates per sample were analyzed.

The EDS analysis was performed at a working distance of 5 mm, with a probe current of 200pA, 2048 channels, process time 6, Acquisition mode: Live time and Acquisition time of 470 live seconds. This analysis focused on visualizing spatial distribution of C, Fe and Si.

Microbial Community Analysis. After using the PowerSoil® RNA and DNA isolation kit, DNA samples were eluted in 50 µl RNase/DNase-Free water. DNA concentrations were determined using a Qubit® 2.0 Fluorometer with DNA HS kit (Life Technologies, Carlsbad, CA, USA). Quantitative PCR (qPCR) specific for the 16S rRNA (gene) of bacteria and archaea was performed as described previously[7]. Microbial 16S rRNA (genes) were amplified using

primers 515F and 806R[8]. Quality and quantity of the purified amplicons were determined using agarose gel electrophoresis and Nanodrop (NanoDrop 1000, Thermo Scientific, Waltham, MA, USA). Subsequent library preparation steps (Nextera, Illumina) and sequencing were performed by Microsynth AG (Switzerland) using the 2 × 250 bp MiSeq Reagent Kit v2 on an Illumina MiSeq sequencing system (Illumina, San Diego, CA, USA). From 113,554 to 151,092 (average 135,126) read pairs were generated per sample. Quality control, reconstruction of 16S rRNA (gene) sequences and taxonomic annotation was performed with nf-core/ampliseq v1.1.2[9, 10] with Nextflow v20.10.0[11] using containerized software with singularity v3.4.2[12]. Primers were trimmed, and untrimmed sequences were discarded (< 13%, on average 9.6%) with Cutadapt v2.6[13]. Adapter and primer-free sequences were imported into QIIME2 version 2019.10.0[14], processed with DADA2 version 1.10.0[15] to eliminate PhiX contamination, trim reads (before the median quality drops below 38, i.e. position 137 in forward reads and 194 in reverse reads), correct errors, merge read pairs, and remove PCR chimeras; ultimately, in total 937 amplicon sequencing variants (ASVs) were obtained across all samples. Alpha rarefaction curves were produced with the QIIME2 diversity alpha-rarefaction plugin, which indicated that the richness of the samples had been fully observed. A Naive Bayes classifier was fitted with 16S rRNA (gene) sequences extracted with the PCR primer sequences from the QIIME compatible, 99%-identity clustered SILVA v132 database[16]. ASVs were classified by taxon using the fitted classifier[17]. 45 ASVs that classified as chloroplasts or mitochondria were removed, totalling to < 0.5% (average 0.36%) relative abundance per sample, and the remaining 892 ASVs had their abundances extracted by feature-table (https://github.com/qiime2/q2-feature-table).

DNA extraction was only successful for the bags deployed in the fully thawed fen until late summer.

Isolation of Fe(III)-reducing bacteria was performed with anoxic media and supplies (5 mM acetate and 5 mM lactate) via multiple rounds of extinction, as previously described[18]. The headspace in the dilution series was N_2:CO_2 (90:10). To the first tube of a dilution series, 1g of FH-coated sand was added, and a 10x dilution series up to a dilution of 10^{-12} was prepared. To identify the isolated Fe(III)-reducing bacteria, DNA was extracted of the isolated culture (after ten transfers) using the UltraClean® Microbial DNA Isolation Kit (MO BIO Laboratories, Carlsbad, CA, US). Then, 16S rRNA gene fragments were amplified using the 341F (CCTACGGGAGGCAGCAG) and 907R (CCGTCAATTCCTTTRAGTTT) primer pair and resulting amplicons were sent for Sanger sequencing (Eurofins GATC biotech, Konstanz, Germany). Sequence results (deposited at database) were analyzed using nucleotide Basic Local Alignment Search Tool (BLAST) to identify the closest relative (performed on the 20th of April 2021, algorithm: blastn, standard database: nucleotide collection (nr/nt), accession number: Y19190.1; see also Figure S5).

Redox Potential Analysis. Redox potential at redox potential probes was recorded every 30 seconds by a datalogger (CR1000, Campbell Scientific) and averaged over 10 minutes. For this study, the reported values at each time point represent the average (± standard error) of values recorded at 6, 8, and 10 cm. In the bog and fen, the values across the two probes were averaged. Redox potential was referenced to two Ag^0/AgCl reference electrodes deployed within the bog and fen. Retrieved data were adjusted to the standard hydrogen electrode (SHE) by adding 213 mV to the recorded values as recommended by the provider company (PaleoTerra).

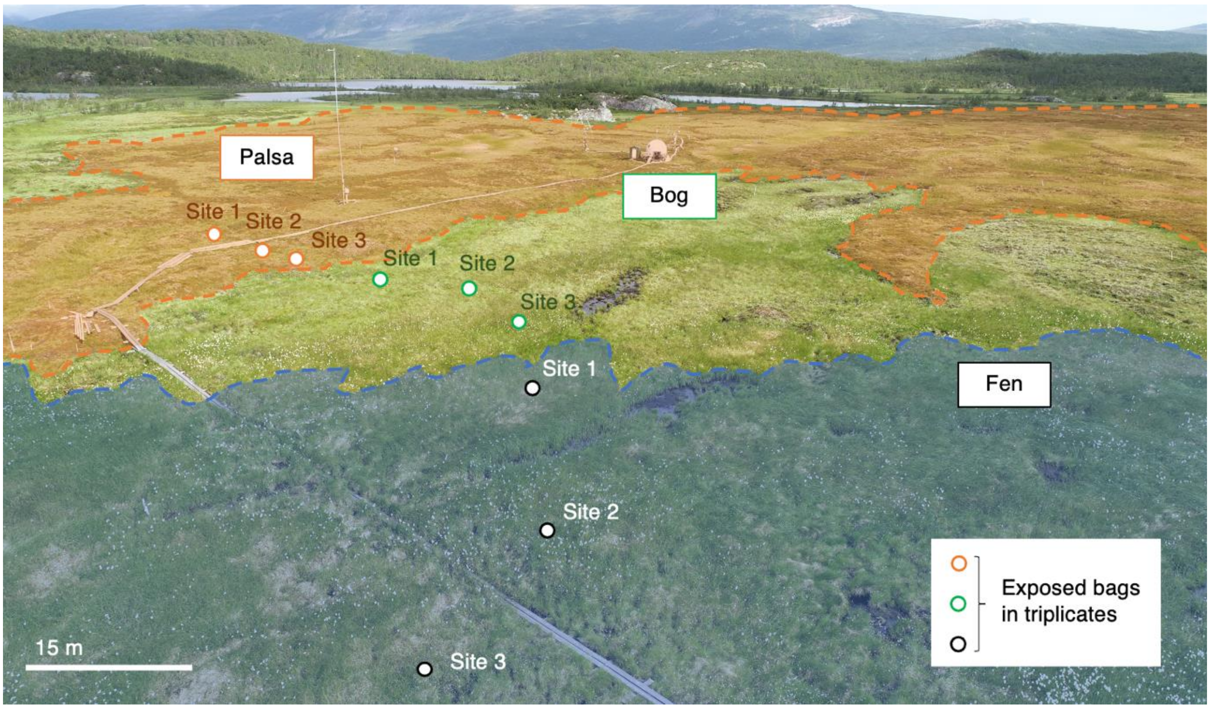

Figure S1. Sites for deployment of the bags with ferrihydrite-coated sand along the thaw gradient from palsa (intact) to bog (partially thawed) to fen (fully thawed) at Stordalen mire, Abisko (Sweden). Orange background marks the palsa, green the bog and blue the fen areas. At each thaw stage (palsa: 68°21'18.91"N, 19° 2'38.02"E to 68°21'18.78"N, 19° 2'39.18"E, bog: 68°21'18.86"N 19° 2'39.94"E to 68°21'18.35"N, 19° 2'40.39"E, fen: 68°21'18.01"N, 19° 2'40.08"E to 68°21'17.38"N, 19° 2'38.99"E), three sites were chosen following the hydrology flow[19]. For the short-term incubation, we deployed three replicate bags of ferrihydrite-coated sand for two weeks during early summer (nine bags per thaw stage in total). For the longer-term incubation of two months, triplicate bags were deployed at the centered positions of each thaw stage to capture conditions characteristic for each thaw stage: palsa site 1, bog site 2 and fen site 2, resulting in three replicate bags per thaw stage.

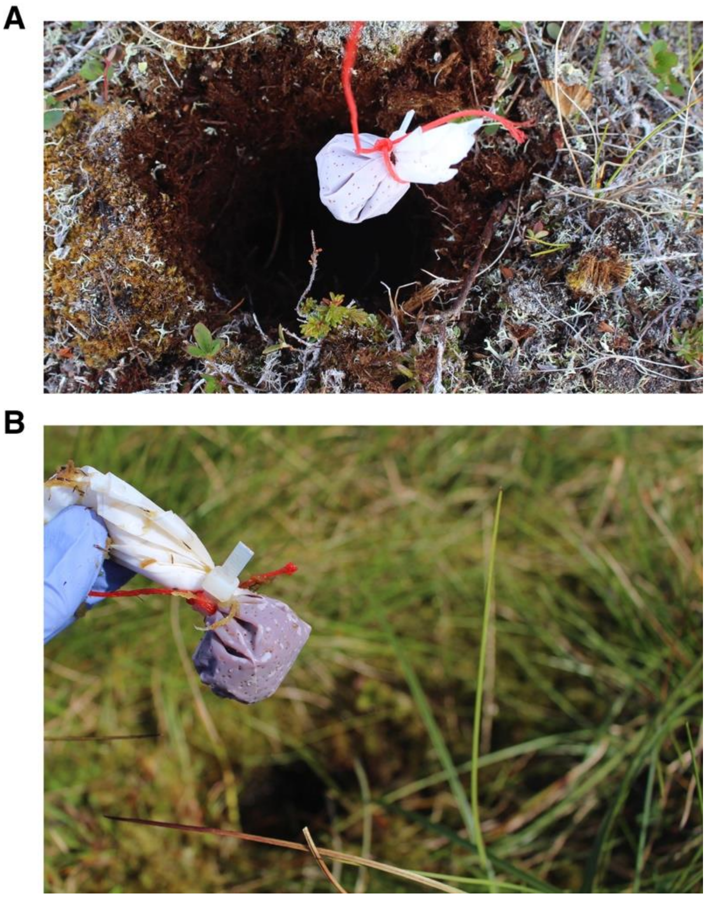

Figure S2. Examples of ferrihydrite-coated sand in teflon bags deployed in intact palsa (A) and fully thawed fen (B). Each bag was placed at 10 cm depth and the hole was re-sealed with the soil layer which had been removed.

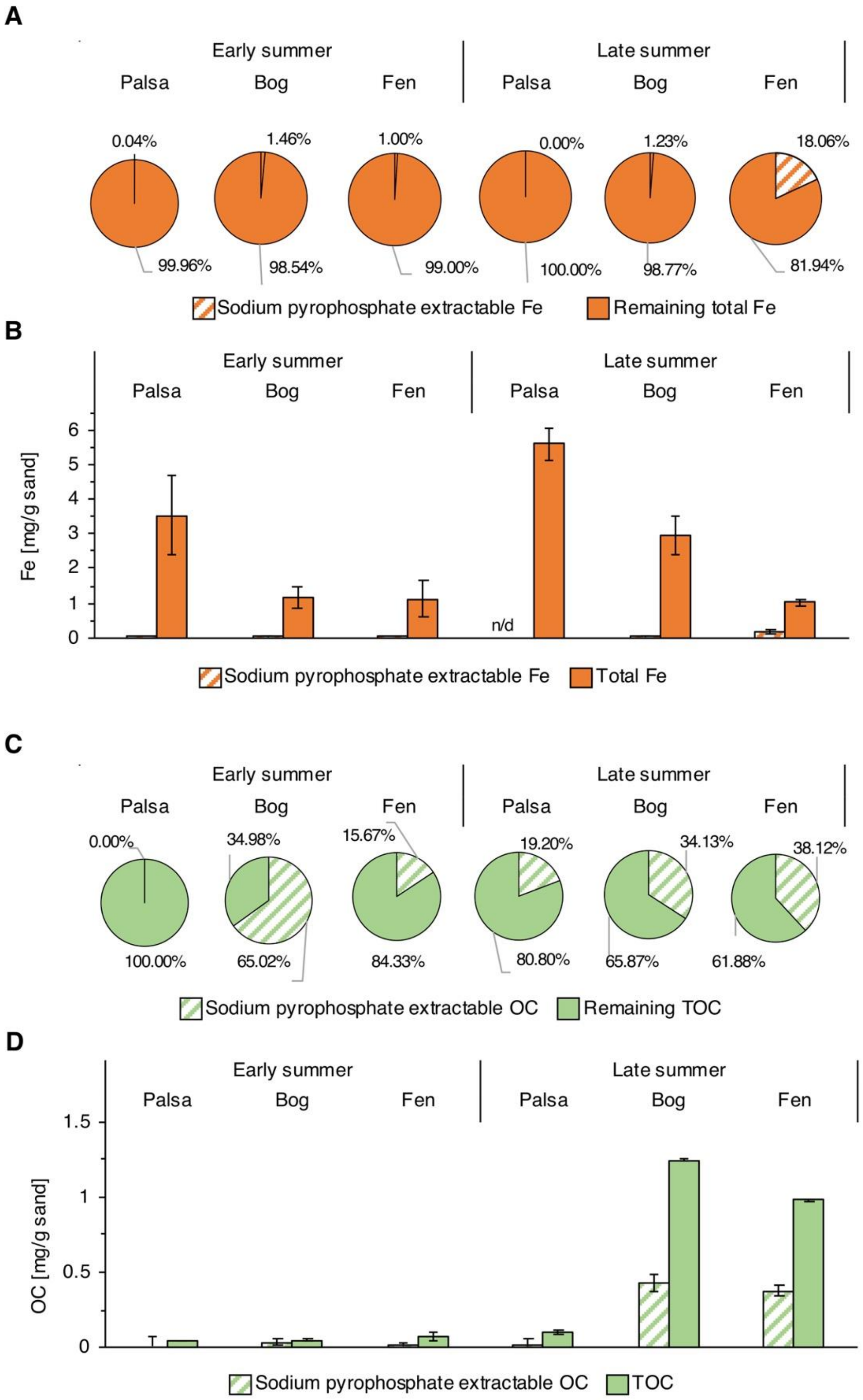
A
Early summer
Late summer
Palsa
Bog
Fen
Palsa
Bog
Fen
0.04%
99.96%
1.46%
98.54%
1.00%
99.00%
0.00%
100.00%
1.23%
98.77%
18.06%
81.94%
Sodium pyrophosphate extractable Fe
Remaining total Fe
B
Early summer
Late summer
Palsa
Bog
Fen
Palsa
Bog
Fen
Fe [mg/g sand]
0
1
2
3
4
5
6
n/d
Sodium pyrophosphate extractable Fe
Total Fe
C
Early summer
Late summer
Palsa
Bog
Fen
Palsa
Bog
Fen
0.00%
100.00%
34.98%
65.02%
15.67%
84.33%
19.20%
80.80%
34.13%
65.87%
38.12%
61.88%
Sodium pyrophosphate extractable OC
Remaining TOC
D
Early summer
Late summer
Palsa
Bog
Fen
Palsa
Bog
Fen
OC [mg/g sand]
0
0.5
1
1.5
Sodium pyrophosphate extractable OC
TOC

Figure S3. Sodium pyrophosphate extractable iron (Fe) and organic carbon (OC) versus total Fe and TOC along the thaw gradient until early summer (2 weeks incubation) and until late summer (2 months incubation). (A) Colloidal/OM-chelated Fe (defined as sodium pyrophosphate extractable Fe) (in orange lines) [% of the total Fe content associated with sand (in orange)] along the thaw gradient with time. (B) Absolute amounts of sodium pyrophosphate extractable Fe and total Fe along the thaw gradient with time. (C) Sodium pyrophosphate extractable OC (in green lines) [% of the TOC associated with sand (in green)] along the thaw gradient with time. (D) Absolute amounts of sodium pyrophosphate extractable OC and TOC along the thaw gradient with time. Reported values for the early summer period are the average and error bars are the standard deviation of triplicate analysis of nine homogenized bags, which were deployed at each thaw stage (palsa, bog and fen). Reported values for the late summer period are the average and error bars are the standard deviation of triplicate analysis of three homogenized bags, deployed at each thaw stage. n/d = not detected.

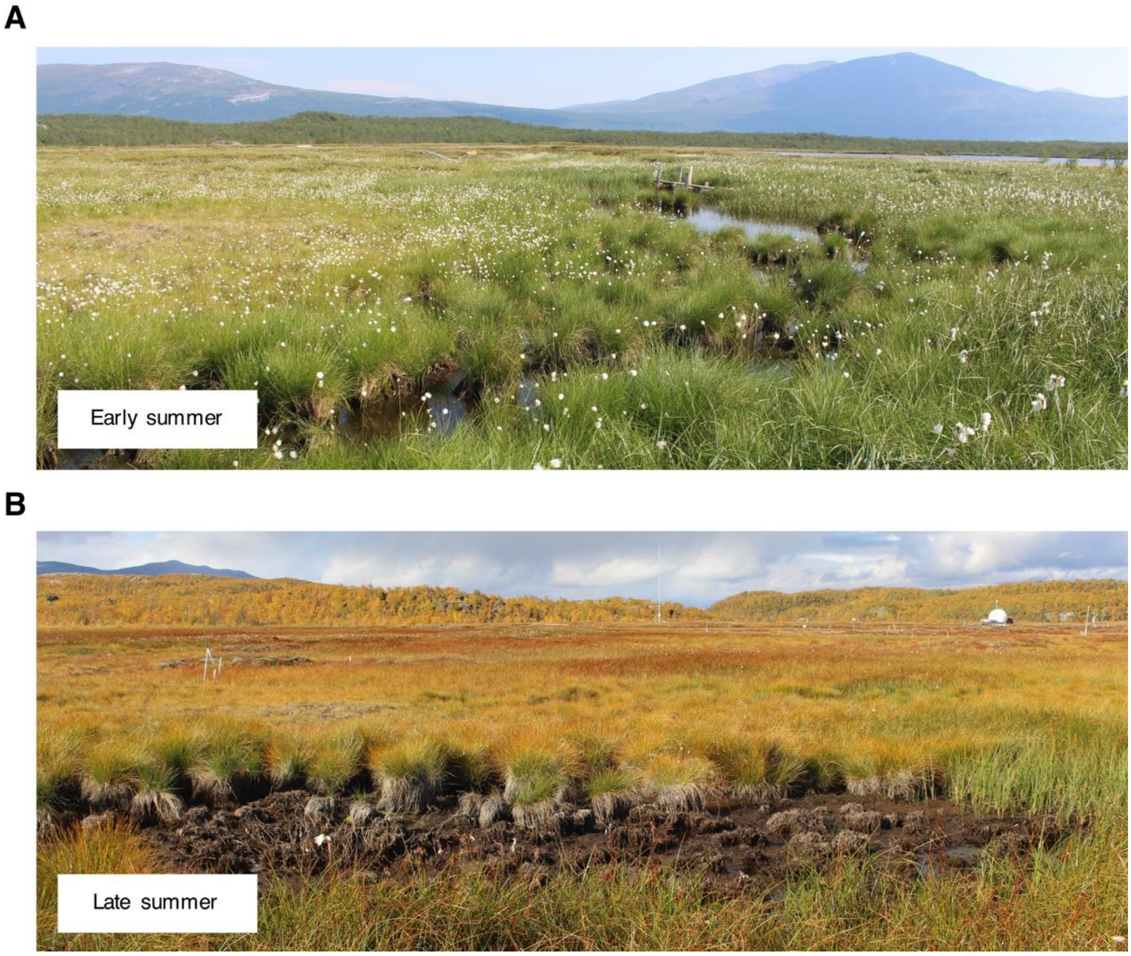

Figure S4. Seasonal changes at Stordalen mire in early summer (A) and late summer (B). In early summer, the semi-wet bog and waterlogged fen areas were completely water-saturated. During summer, bog areas became drier due to increasing drainage caused by active layer deepening and decreasing volumetric soil water content in the upper 10 cm.

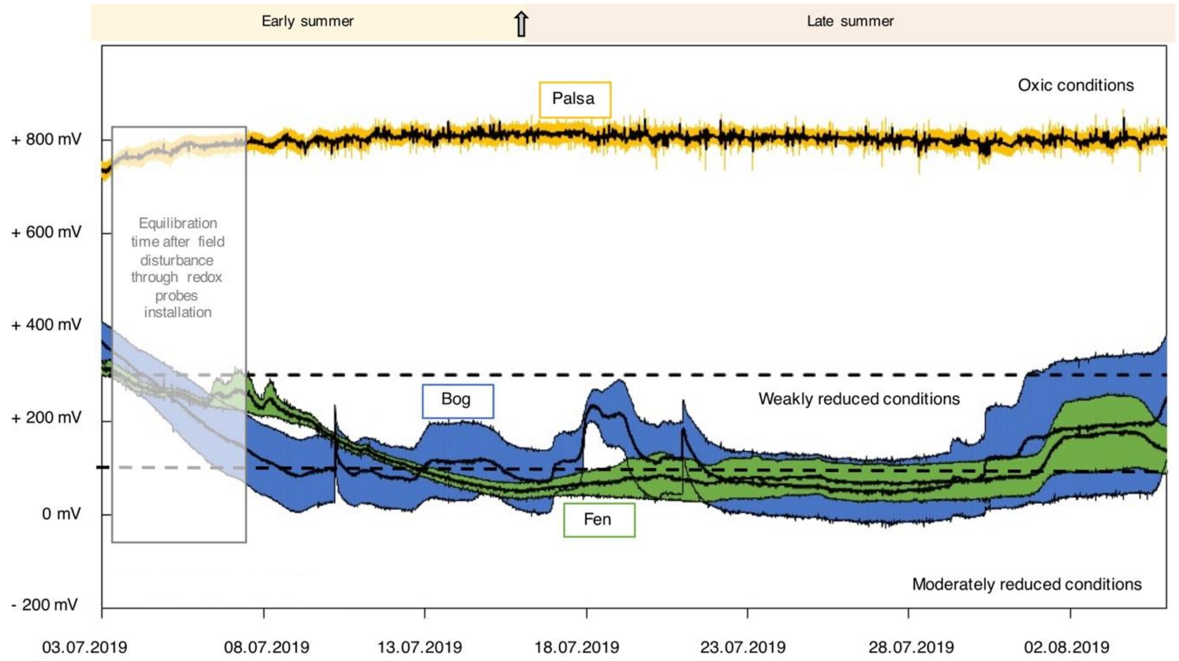

Figure S5. Redox potential along the thaw gradient. Black lines report the average redox potential [mV] and the colored areas represent the standard deviation across the three depths at 6, 8 and 10 cm in each thaw stage. The redox potential measured in palsa is marked in yellow, in bog in blue and in fen in green. The first 1-2 days represent the equilibrium phase after installing the redox probes in the field. Values above +300 mV are considered as oxic conditions. Values of +300 mV to +100 mV are considered as weakly reduced and values of +100 to -100 mV as moderately reducing conditions[20]. Towards August, the redox potential in bog increases from 0 mV to above +300 mV which marks a potential shift from Fe(III)-reducing to Fe(II)-oxidizing conditions. Unfortunately, the redox probes disconnected from the battery in mid-August and remote data collection ceased.

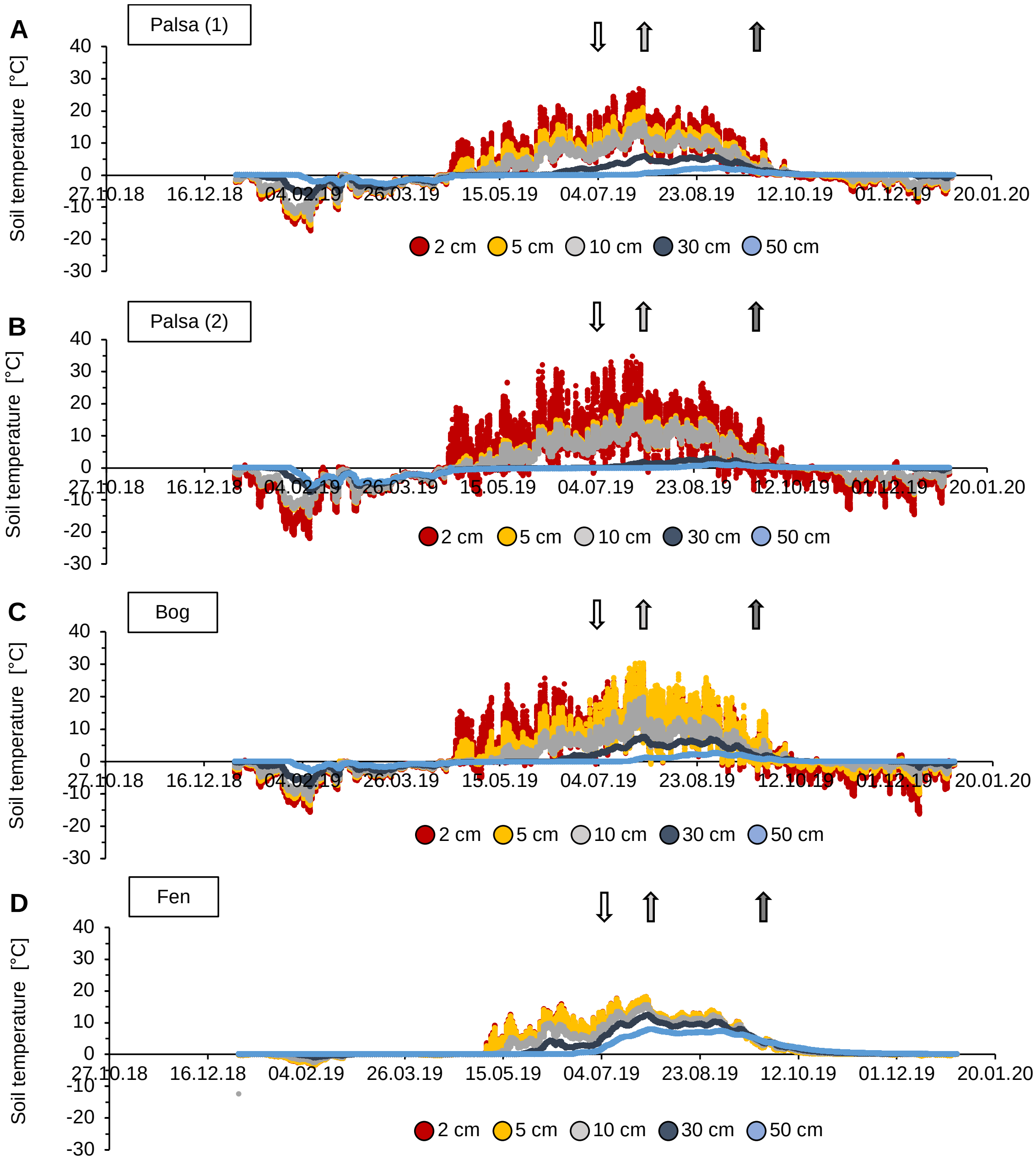

Figure S6. Soil temperatures at each thaw stage at 2 cm, 5 cm, 10 cm, 30 cm and 50 cm soil depth: (A) palsa (replicate 1; 68°21'22.25"N, 19° 2'42.22"E), (B) palsa (replicate 2; 68°21'21.05"N, 19° 2'38.85"E), (C) bog (68°21'20.49"N, 19° 2'45.52"E) and (D) fen (68°21'20.20"N, 19° 2'42.37"E) in the year 2019[21].

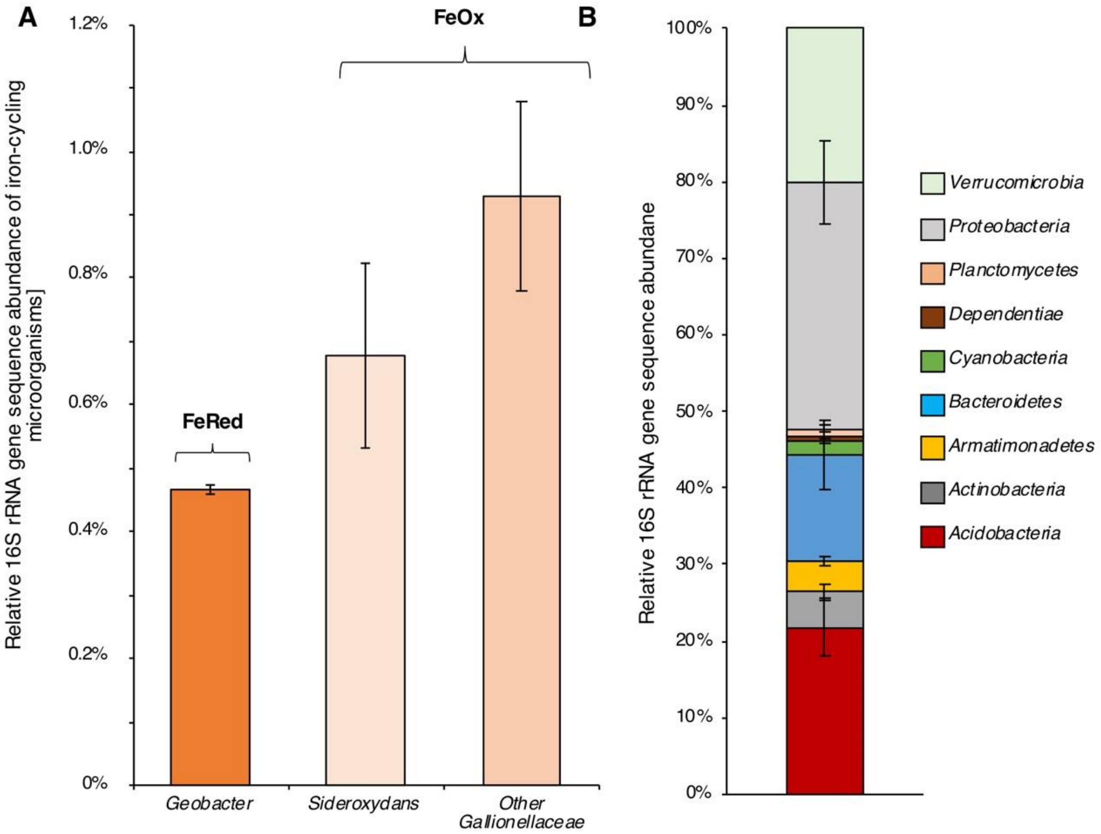

Figure S7. Microbial community of ferrihydrite-coated sand deployed for 2 months in the fully thawed fen until late summer. (A) Detected microbial iron-metabolizing key players: Fe(III)-reducing bacteria (FeRed) and Fe(II)-oxidizing bacteria (FeOx). (B) Present microbial community. Values and error bars represent average and standard deviation of triplicate 16S rRNA gene amplicon sequencing abundance analysis of homogenized ferrihydrite-coated sand that was incubated in the fully thawed fen from early to late summer.

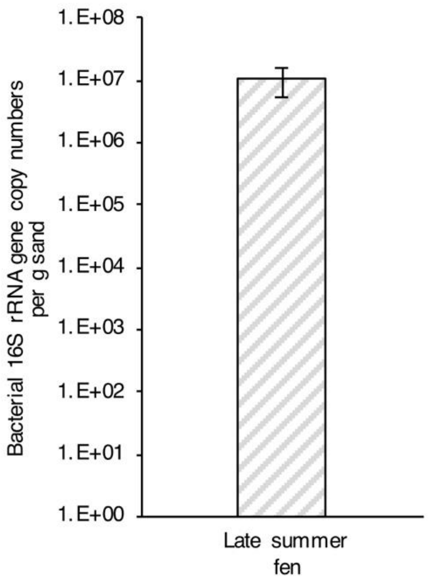

Figure S8. Bacterial 16S rRNA gene copy numbers based on qPCR analysis of the homogenized ferrihydrite-coated sand incubated for 2 months in the fully thawed fen until late summer.

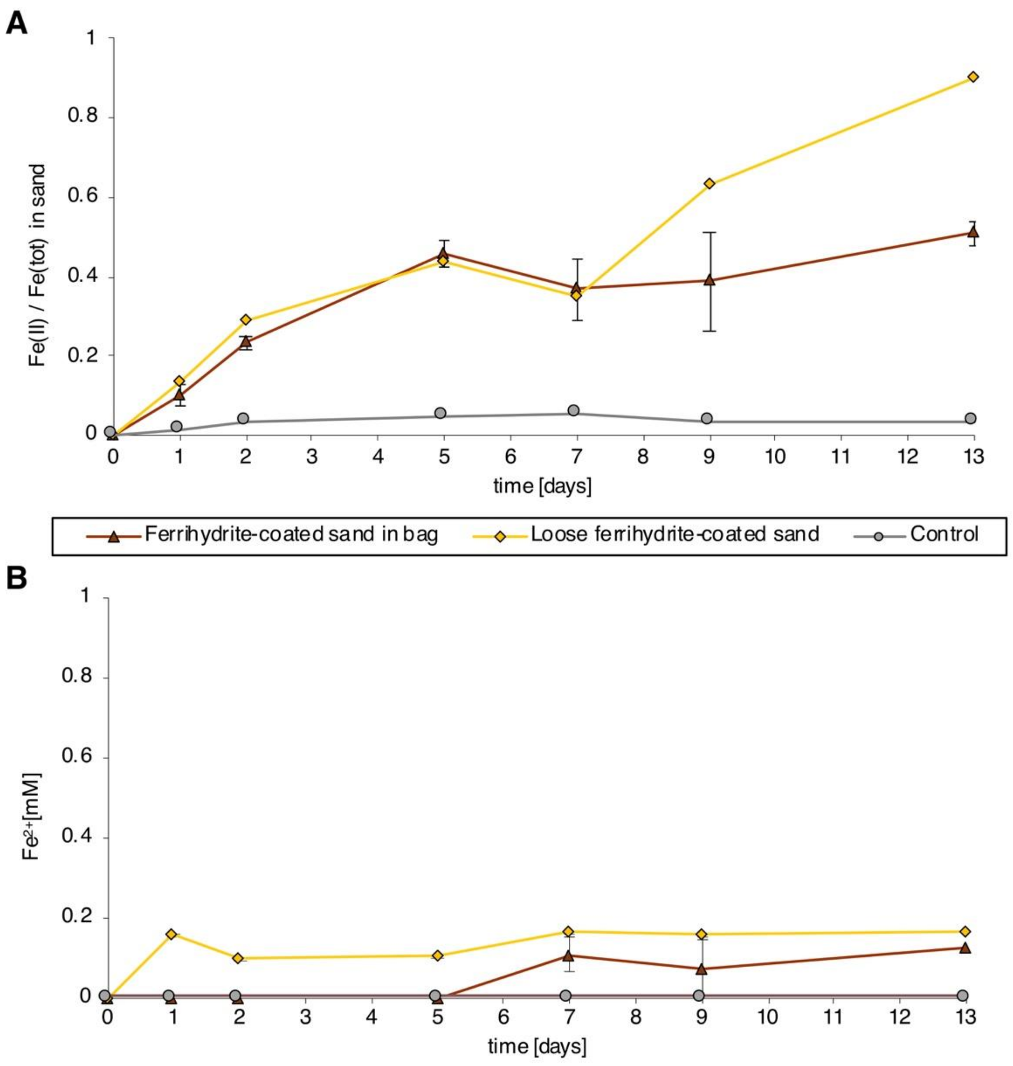

Figure S9. Microbial Fe(III) reduction under lab conditions in loose ferrihydrite-coated sand versus ferrihydrite-coated sand in teflon bags. (A) Fe(II)/Fe(tot) ratio in sand over 13 days in anoxic fresh water media, containing 5 mM acetate, 2 mM cysteine and inoculated with *Geobacter metallidurans* (10^8 cells/mL). Reported values represent the average and range of duplicates of each setup. Yellow shows the Fe(II)/Fe(tot) ratio during reduction of loose ferrihydrite-coated sand over time. Brown shows the Fe(II)/Fe(tot) ratio in setups with ferrihydrite-coated sand in teflon bags over time. Control represents data for incubation of loose ferrihydrite-coated sand without *Geobacter metallidurans* inoculum. An Fe(II)/Fe(tot) ratio of 1 means 100% Fe(II) formation in the sand phase. (B) Only low concentrations of dissolved Fe^{2+} (below 0.2 mM) were present in the liquid phase.

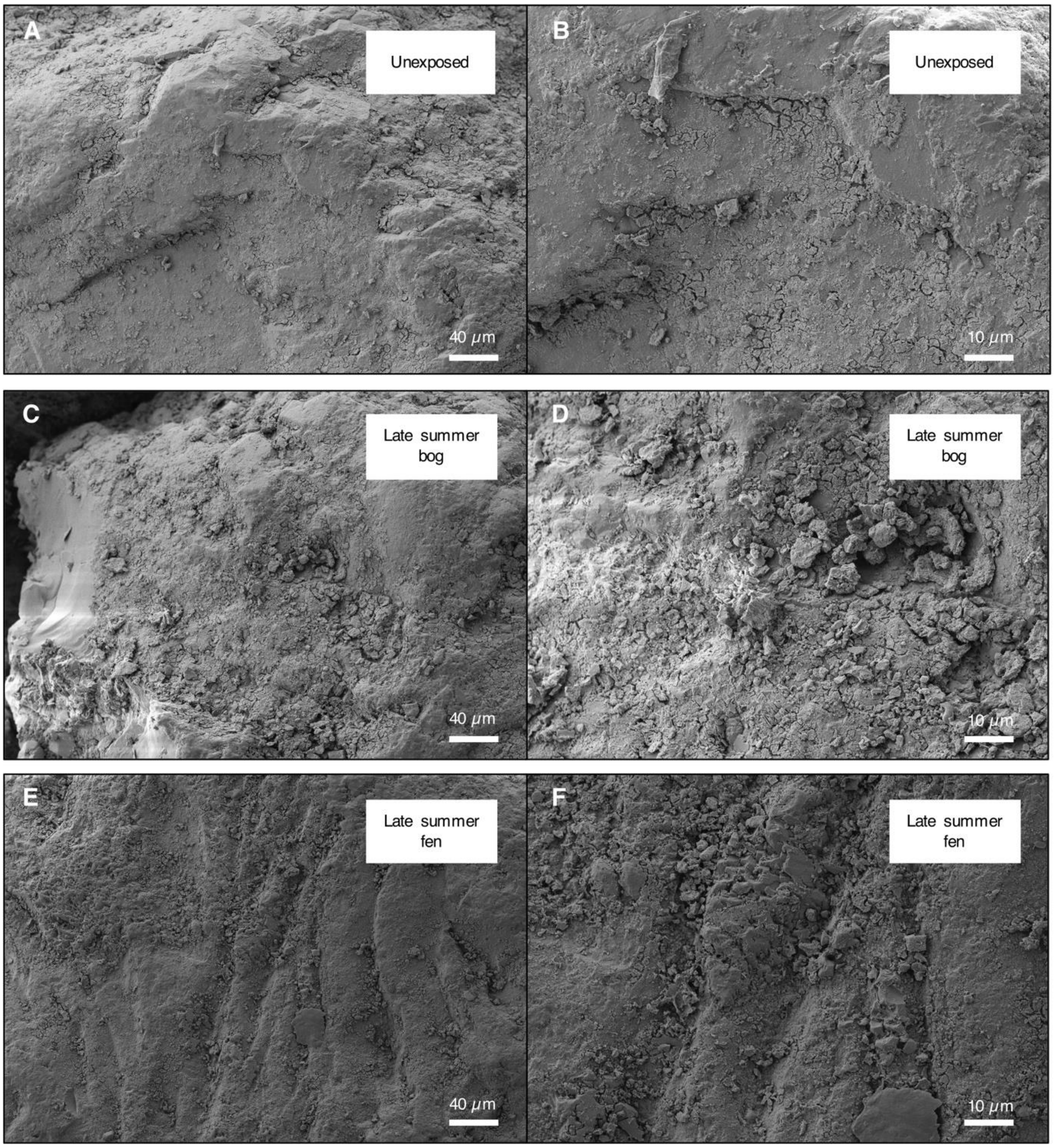

Figure S10. Scanning electron microscopy surface analysis of the ferrihydrite-coated sands before and after incubation. Unexposed sand shows smooth surfaces and small aggregate formation on the sand grains: (A) overview image and (B) close up image of unexposed sand. Ferrihydrite-coated sand from bags deployed for 2 months until late summer in the partially thawed bog shows aggregate formation on the surface of the sand grains: (C) overview image and (D) close up image of sand from bags deployed for 2 months until late summer in the partially thawed bog (replicate analysis to Figure 4 shown in the main text). Ferrihydrite-coated sand from bags deployed for 2 months until late summer in the fully thawed fen shows aggregate formation on the sand grains surface: (E) overview image and (F) close up image of sand from bag deployed until late summer in the fully thawed fen.

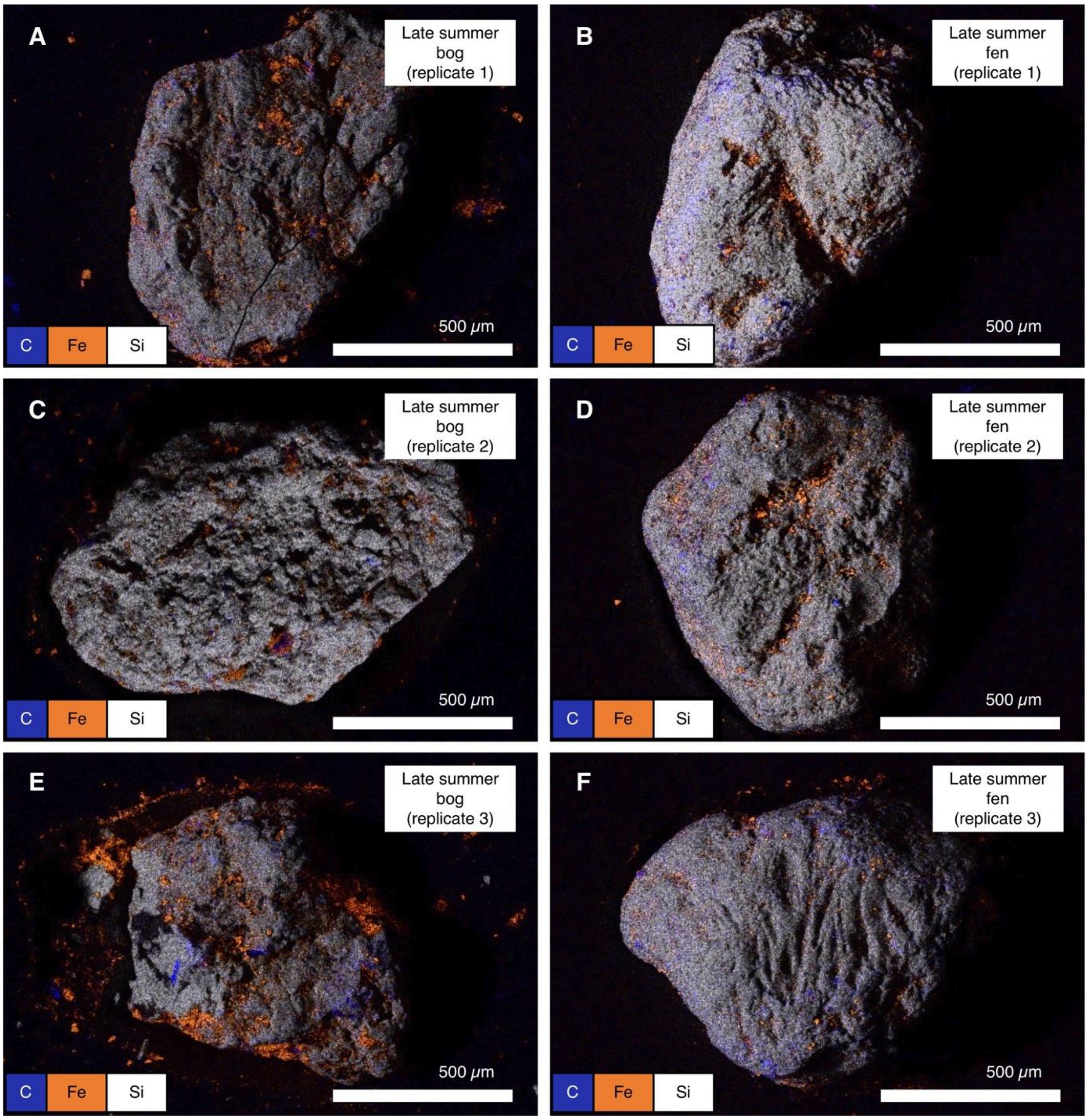

Figure S11. EDS derived chemical distribution maps of the replicate analysis of iron (Fe)-organic carbon (OC) associations at the surface of the ferrihydrite-coated sand grains from bags deployed in partially thawed bog and fully thawed fen collected after incubation for 2 months (late summer): (A) and (B) replicate 1, (C) and (D) replicate 2, (E) and (F) replicate 3.

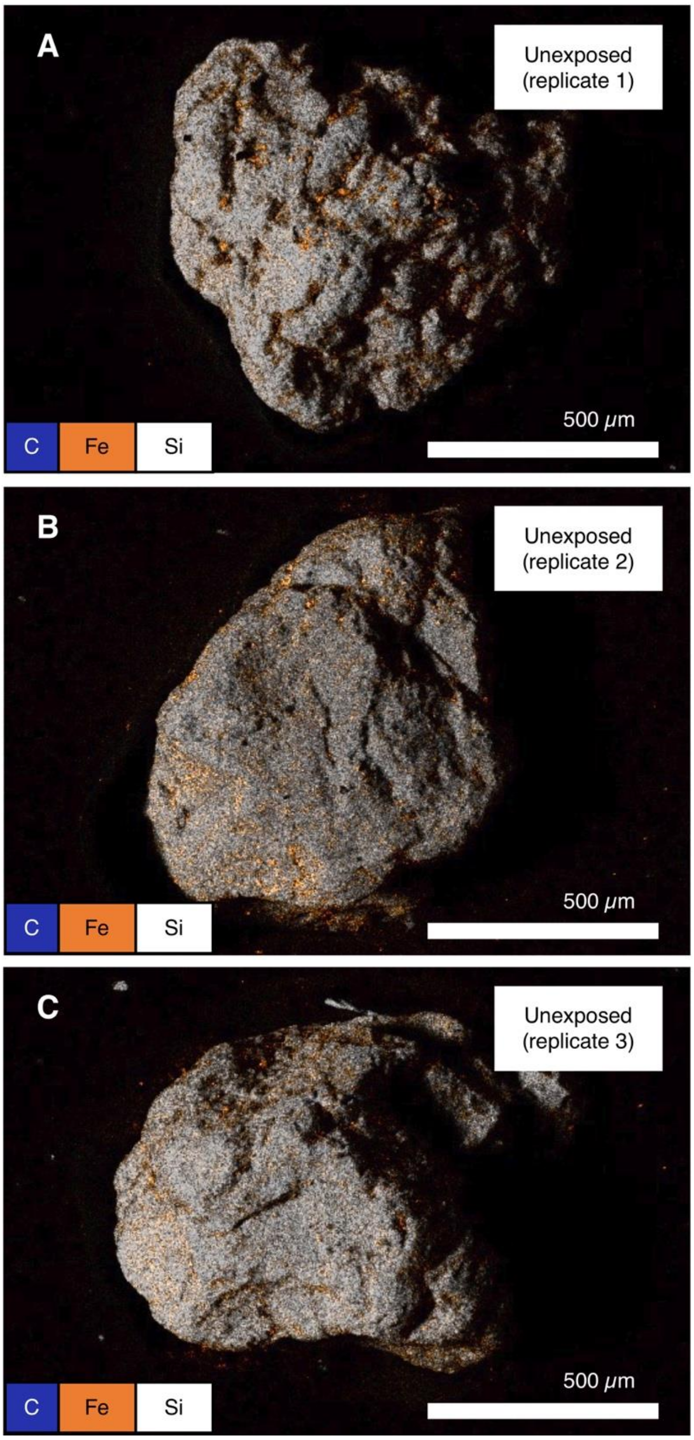

Figure S12. EDS derived chemical distribution maps of iron (Fe) coating on unexposed ferrihydrite-coated sand grains: (A) replicate 1, (B) replicate 2 and (C) replicate 3.

Table S1. Organic carbon (OC) to iron (Fe) ratios in the active layer of the partially thawed bog and in the fully thawed fen collected after incubation of 2 months until late summer.

		**Total extractable OC and Fe**	**Fe-bound OC***
		TOC/totalFe (wt:wt)	OC/Fe (wt:wt)
Late summer	Bog	0.42	0.28
	Fen	0.96	0.73
		TOC/total Fe (molar ratio)	OC/Fe (molar ratio)
Late summer	Bog	1.96	1.31
	Fen	4.47	3.38

*(TOC-sodiumpyrophosphate extractable OC)/(total Fe-sodium pyrophosphate extractable Fe)

References:

1. Sorwat, J.; Mellage, A.; Kappler, A.; Byrne J. M. Immobilizing magnetite onto quartz sand for chromium remediation. *Journal of Hazardous Materials* **2020,** *400*, 123139.

2. Kango, S.; Kumar, R. Magnetite nanoparticles coated sand for arsenic removal from drinking water. *Environ Earth Sci* **2016,** *75*, (5), 381.

3. Stookey, L. L. Ferrozine - a New Spectrophotometric Reagent for Iron. *Anal Chem* **1970,** *42*, (7), 779-781.

4. Biswas, S.; Vijayan, K. Friction and wear of ptfe - a review. *Wear* **1992,** *158*, 193-211.

5. Buerkle, M.; Asai, Y. Thermal conductance of teflon and polyethylene: insight from an atomistic, single-molecule level. *Sci Rep* **2017,** *7,* 41898 .

6. Lueder, U.; Maisch, M.; Laufer, K.; Jorgensen, B.B.; Kappler, A.; Schmidt, C. Influence of physical pertubation on Fe(II) supply in coastal marine sediments. *Environ Sci Technol* **2020,** *54*, 3209-3218.

7. Otte, J. M.; Harter, J.; Laufer, K.; Blackwell, N.; Straub, D.; Kappler, A.; Kleindienst, S. The distribution of active iron-cycling bacteria in marine and freshwater sediments is decoupled from geochemical gradients. *Method Enzymol* **2018,** *20*, (7), 2483-2499.

8. Caporaso, J. G.; Kuczynski, J.; Stombaugh, J.; Bittinger, K.; Bushman, F. D.; Costello, E. K.; Fierer, N.; Pena, A. G.; Goodrich, J. K.; Gordon, J. I.; Huttley, G. A.; Kelley, S. T.; Knights, D.; Koenig, J. E.; Ley, R. E.; Lozupone, C. A.; McDonald, D.; Muegge, B. D.; Pirrung, M.; Reeder, J.; Sevinsky, J. R.; Tumbaugh, P. J.; Walters, W. A.; Widmann, J.; Yatsunenko, T.; Zaneveld, J.; Knight, R. QIIME allows analysis of high-throughput community sequencing data. *Nat Methods* **2010,** *7,* (5), 335-336.

9. Ewels, P. A.; Peltzer, A.; Fillinger, S.; Patel, H.; Alneberg, J.; Wilm, A.; Garcia, M. U.; Di Tommaso, P.; Nahnsen, S. The nf-core framework for community-curated bioinformatics pipelines. *Nat Biotechnol* **2020,** *38*, (3), 276-278.

10. Straub, D.; Blackwell, N.; Langarica-Fuentes, A.; Peltzer, A.; Nahnsen, S.; Kleindienst, S. Interpretations of environmental microbial community studies are biased by the selected 16S rRNA (gene) amplicon sequencing pipeline. *Front Microbiol* **2020,** *11*, 550420.

11. Di Tommaso, P.; Chatzou, M.; Floden, E. W.; Barja, P. P.; Palumbo, E.; Notredame, C. Nextflow enables reproducible computational workflows. *Nat Biotechnol* **2017,** *35*, (4), 316-319.

12. Kurtzer, G. M.; Sochat, V.; Bauer, M. W. Singularity: Scientific containers for mobility of compute. *PLoS One* **2017,** *12*, (5), e0177459.

13. Martin, M. Cutadapt removes adapter sequences from high-throughput sequencing reads. *EMBnet journal* **2011,** *17*, (1), 3.

14. Bolyen, E.; Rideout, J. R.; Dillon, M. R.; Bokulich, N. A.; Abnet, C. C.; Al-Ghalith, G. A.; Alexander, H.; Alm, E. J.; Arumugam, M.; Asnicar, F.; Bai, Y.; Bisanz, J. E.; Bittinger, K.; Brejnrod, A.; Brislawn, C. J.; Brown, C. T.; Callahan, B. J.; Caraballo-Rodríguez, A. M.; Chase,

J.; Cope, E. K.; Da Silva, R.; Diener, C.; Dorrestein, P. C.; Douglas, G. M.; Durall, D. M.; Duvallet, C.; Edwardson, C. F.; Ernst, M.; Estaki, M.; Fouquier, J.; Gauglitz, J. M.; Gibbons, S. M.; Gibson, D. L.; Gonzalez, A.; Gorlick, K.; Guo, J.; Hillmann, B.; Holmes, S.; Holste, H.; Huttenhower, C.; Huttley, G. A.; Janssen, S.; Jarmusch, A. K.; Jiang, L.; Kaehler, B. D.; Kang, K. B.; Keefe, C. R.; Keim, P.; Kelley, S. T.; Knights, D.; Koester, I.; Kosciolek, T.; Kreps, J.; Langille, M. G. I.; Lee, J.; Ley, R.; Liu, Y. X.; Loftfield, E.; Lozupone, C.; Maher, M.; Marotz, C.; Martin, B. D.; McDonald, D.; McIver, L. J.; Melnik, A. V.; Metcalf, J. L.; Morgan, S. C.; Morton, J. T.; Naimey, A. T.; Navas-Molina, J. A.; Nothias, L. F.; Orchanian, S. B.; Pearson, T.; Peoples, S. L.; Petras, D.; Preuss, M. L.; Pruesse, E.; Rasmussen, L. B.; Rivers, A.; Robeson, M. S., 2nd; Rosenthal, P.; Segata, N.; Shaffer, M.; Shiffer, A.; Sinha, R.; Song, S. J.; Spear, J. R.; Swafford, A. D.; Thompson, L. R.; Torres, P. J.; Trinh, P.; Tripathi, A.; Turnbaugh, P. J.; Ul-Hasan, S.; van der Hooft, J. J. J.; Vargas, F.; Vázquez-Baeza, Y.; Vogtmann, E.; von Hippel, M.; Walters, W.; Wan, Y.; Wang, M.; Warren, J.; Weber, K. C.; Williamson, C. H. D.; Willis, A. D.; Xu, Z. Z.; Zaneveld, J. R.; Zhang, Y.; Zhu, Q.; Knight, R.; Caporaso, J. G. Reproducible, interactive, scalable and extensible microbiome data science using QIIME 2. *Nat Biotechnol* **2019,** *37*, (8), 852-857.

15. Callahan, B. J.; McMurdie, P. J.; Rosen, M. J.; Han, A. W.; Johnson, A. J.; Holmes, S. P. DADA2: High-resolution sample inference from Illumina amplicon data. *Nat Methods* **2016,** *13*, (7), 581-3.

16. Pruesse, E.; Quast, C.; Knittel, K.; Fuchs, B. M.; Ludwig, W.; Peplies, J.; Glockner, F. O. SILVA: a comprehensive online resource for quality checked and aligned ribosomal RNA sequence data compatible with ARB. *Nucleic Acids Res* **2007,** *35*, (21), 7188-96.

17. Bokulich, N. A.; Kaehler, B. D.; Rideout, J. R.; Dillon, M.; Bolyen, E.; Knight, R.; Huttley, G. A.; Gregory Caporaso, J. Optimizing taxonomic classification of marker-gene amplicon sequences with QIIME 2's q2-feature-classifier plugin. *Microbiome* **2018,** *6*, (1), 90.

18. Patzner, M. S.; Mueller, C. W.; Malusova, M.; Baur, M.; Nikeleit, V.; Scholten, T.; Hoeschen, C.; Byrne, J. M.; Borch, T.; Kappler, A.; Bryce, C. Iron mineral dissolution releases iron and associated organic carbon during permafrost thaw. *Nat Commun* **2020**,11, 6329.

19. Olefeldt, D.; Roulet, N. T. Effects of permafrost and hydrology on the composition and transport of dissolved organic carbon in a subarctic peatland complex. *J Geophys Res-Biogeo* **2012,** *117,* G01005.

20. Reddy, K. R.; DeLaune, R.D. Biogeochemistry of wetlands: Science and applications. *CRC Press, Boca Raton, FL.* **2008**, ISBN 978-1-56670-678-0.

21. Rinne, J., ICOS Sweden Ecosystem eco time series (ICOS Sweden), Abisko-Stordalen Palsa Bog, 2018-12-31–2019-12-31, https://hdl.handle.net/11676/s5oBzukX_FaXpHU___86QasO) **2021**.
